# The contribution of BvgR, RisA, and RisS to global gene regulation, intracellular cyclic-di-GMP levels, motility, and biofilm formation in *Bordetella bronchiseptica*

**DOI:** 10.3389/fmicb.2024.1305097

**Published:** 2024-03-07

**Authors:** Tracy L. Nicholson, Ursula Waack, Damarius S. Fleming, Qing Chen, Laura C. Miller, Tod J. Merkel, Scott Stibitz

**Affiliations:** ^1^Agricultural Research Service, USDA, National Animal Disease Center, Ames, IA, United States; ^2^United States Department of Energy, Oak Ridge Institute for Science and Education, Oak Ridge, TN, United States; ^3^USDA, Agricultural Research Service, Beltsville Agricultural Research Center, Beltsville, MD, United States; ^4^Division of Bacterial, Parasitic, and Allergenic Products, Center for Biologics Evaluation and Research, FDA, Silver Spring, MD, United States; ^5^Department of Diagnostic Medicine/Pathobiology, College of Veterinary Medicine, Kansas State University, Manhattan, KS, United States

**Keywords:** *Bordetella bronchiseptica*, RNA-seq, cyclic-di-GMP, motility, biofilm

## Abstract

*Bordetella bronchiseptica* is a highly contagious respiratory bacterial veterinary pathogen. In this study the contribution of the transcriptional regulators BvgR, RisA, RisS, and the phosphorylation of RisA to global gene regulation, intracellular cyclic-di-GMP levels, motility, and biofilm formation were evaluated. Next Generation Sequencing (RNASeq) was used to differentiate the global gene regulation of both virulence-activated and virulence-repressed genes by each of these factors. The BvgAS system, along with BvgR, RisA, and the phosphorylation of RisA served in cyclic-di-GMP degradation. BvgR and unphosphorylated RisA were found to temporally regulate motility. Additionally, BvgR, RisA, and RisS were found to be required for biofilm formation.

## Introduction

*Bordetella bronchiseptica* is a highly contagious, gram-negative, bacterial veterinary pathogen. It can serve as the primary etiologic agent or as a co-contributor to a spectrum of clinical disease outcomes ranging from asymptomatic carriage to severe bronchopneumonia (Mattoo and Cherry, 2005; Brockmeier et al., 2019; Chambers et al., 2019). *B. bronchiseptica* is closely related to *Bordetella pertussis* and *Bordetella parapertussis*, the causative agents of whooping cough in humans (Goodnow, 1980; Parkhill et al., 2003; Preston et al., 2004; Diavatopoulos et al., 2005). In fact, data suggest that *B. pertussis* and *B. parapertussis* evolved from a *B. bronchiseptica* ancestor (Parkhill et al., 2003; Preston et al., 2004; Diavatopoulos et al., 2005). In addition to their close genetic relatedness, classical *Bordetella* species harbor many of the same virulence factors, which are similarly regulated (Parkhill et al., 2003; Linz et al., 2016; Chen and Stibitz, 2019). Despite these similarities, the classical *Bordetella* species differ in traits such as host specificity, disease severity, and duration of infection. *B. pertussis* only infects humans, lacks an animal reservoir, and lacks the ability to survive in the environment (Hewlett et al., 2014; Pinto and Merkel, 2017). In contrast, *B. bronchiseptica* infects a variety of animals, often establishing chronic infections that range from lethal pneumonia to asymptomatic carriage and is capable of surviving in the environment (Goodnow, 1980; Brockmeier et al., 2019). While rare and more frequent in immunocompromised individuals, human infections have been reported (Register et al., 2012; Agarwal et al., 2022; Barcala Salido et al., 2022; Moore et al., 2022).

A key regulatory mechanism is shared among the classical *Bordetella* species in that the majority of virulence gene expression is regulated by a two-component sensory transduction system encoded by the *bvg* locus. This locus comprises a histidine kinase sensor protein, BvgS, and a DNA-binding response-regulator protein, BvgA. In response to environmental cues, such as temperature, MgSO_4_, or nicotinic acid concentrations, BvgAS controls expression of a spectrum of phenotypic phases transitioning between a virulent (Bvg+) phase and a non-virulent (Bvg-) mode, a process referred to as phenotypic modulation. During the virulent Bvg^+^ phase, the BvgAS system is fully active and virulence-activated genes (vags), such as filamentous hemagglutinin (FHA), pertactin (PRN), fimbriae, adenylate cyclase toxin (ACT), and a type III secretion system (T3SS), are fully expressed (Cotter and Jones, 2003). Conversely, BvgAS is inactive during the Bvg-phase, resulting in the maximal expression of motility loci and virulence-repressed genes (*vrg* genes) (Akerley et al., 1992, 1995; McMillan et al., 1996).

Two genes, *bvgR* and *risA,* have been demonstrated to be involved in the regulation of vrgs (Merkel and Stibitz, 1995; Croinin et al., 2005). BvgR is a vag and functions as a repressor to vrg genes during non-modulating (Bvg+) conditions (Merkel and Stibitz, 1995; Merkel et al., 1998). RisA is a member of the OmpR family of two-component response regulators and has been demonstrated to be required for maximal expression of at least some vrgs (Croinin et al., 2005; Stenson et al., 2005). The *risA* gene is adjacent to *risS* encoding a sensor kinase of the RisA/S two-component system (Chen et al., 2017). *risA* was originally identified and named from a *B. bronchiseptica* transposon mutagenesis screen as a gene required for survival (reduction in intracellular survival) (Jungnitz et al., 1998). While the regulatory mechanism of action for BvgR is not fully understood, it shares sequence similarity to diguanylate phosphodiesterases and contains a conserved EAL sequence found in diguanylate phosphodiesterases that are involved in the degradation of bis-(3′-5′)-cyclic-dimeric guanosine monophosphate (c-di-GMP). c-di-GMP is a bacterial second messenger known to regulate a variety of cellular processes and has been shown to promote biofilm formation and inhibit motility for *B. bronchiseptica* (Sisti et al., 2013).

The global transcriptional response orchestrated by BvgR and RisA in *B. pertussis* has recently been reported (Coutte et al., 2016). In this study we utilize RNA-sequencing to evaluate the global transcriptome of wildtype *B. bronchiseptica*, *ΔbvgR*, *ΔrisA*, *risAD60N*, and *ΔrisAS* during both modulating and non-modulating conditions. We additionally assess the contribution of BvgR, RisA, phosphorylation of RisA, and RisS to intracellular c-di-GMP and GMP levels, motility, and biofilm formation. Given the ancestral link between *B. pertussis* and *B. bronchiseptica*, the data reported here provides an evolutionary perspective on gene regulation and virulence phenotypes.

## Materials and methods

### Bacterial strains and culture conditions

The bacterial strains and plasmids used in this study are listed in Table 1. *E. coli* strains were grown in Lysogeny Broth (LB) medium or on Lysogeny Agar (LA) plates. All *B. bronchiseptica* strains were grown at 37^°^ C on Bordet-Gengou (BG) agar (Difco, Sparks, MD) supplemented with 10% sheep’s blood or in Stainer-Scholte (SS) broth. When appropriate, antibiotics were included at the following concentrations: carbenicillin, 100 μg/mL; kanamycin, 100 μg/mL; streptomycin, 40 μg/mL; chloramphenicol, 30 μg/mL; gentamicin, 100 μg/mL; and 200 ug/mL 2, 6-diaminopimelic acid (DAP). BG agar or SS broth supplemented with 50 mM MgSO4 was used to induce the Bvg-mode of *B. bronchiseptica* strains.

**Table 1:** Bacterial strains and plasmids used in this study.

Strain or plasmid	Relevant feature(s)	Source or references
Strains		
*E. coli*		
MFD*pir*	MG1655 RP4-2-Tc*::*[*ΔMu1::aac(3)IV-ΔaphA-Δnic35-ΔMu2::zeo*] *ΔdapA::(erm-pir) ΔrecA* (Apra^R^ Zeo^R^ Erm^R^)	Ferrieres et al. (2010)
RHO3	SM10(*lpir*)*Δasd::FRT ΔaphA::FRT* (Km^S^)	Lopez et al. (2009)
*B. bronchiseptica*		
KM22		Nicholson et al. (2020)
TN23	KM22 bvgS-C3 (bvgSR570H) Bvg+	Nicholson et al. (2012a)
TN30	KM22 *ΔbvgS* Bvg-	Nicholson et al. (2012a)
TN47	KM22 *ΔbvgR*	This study
QC5012	KM22 *ΔrisA*	This study
QC5010	KM22 *ΔrisA::CM*, CM^R^	This study
QC5013	KM22 *ΔrisS*	This study
QC5011	KM22 *ΔrisS::CM*, CM^R^	This study
QC5014	KM22 *ΔrisAS*	This study
QC5009	KM22 *ΔrisAS::CM*, CM^R^	This study
QC5402	KM22 risAD60N	This study
Plasmids		
pSS4245	Allelic exchange vector, *oriT ColEl oriV* P*ptx*-I-SceI (Carb^R^ Kan^R^)	Buboltz et al. (2009) and Bone et al. (2017)
pSS4894	Allelic exchange vector, *oriT ColEl oriV* P*ptx*-I-SceI (Gent^R^)	Chen et al. (2017)
pTN90	pSS4245::*ΔbvgR*, Carb^R^ Kan^R^	This study
pQC2429	pSS4894::*ΔrisA*::CM, CM^R^,Gent^R^	This study
pQC2428	pSS4894::*ΔrisA*, Gent^R^	This study
pQC2430	pSS4894::*ΔrisS*::CM, CM^R^,Gent^R^	This study
pQC2417	pSS4894::*ΔrisS*, Gent^R^	This study
pQC2427	pSS4894::*ΔrisAS*::CM, CM^R^,Gent^R^	This study
pQC2426	pSS4894::*ΔrisAS*, Gent^R^	This study
pSS5083	pSS4894::risAD60N, Gent^R^	This study

### Construction of *Bordetella bronchiseptica* mutant strains harboring in-frame deletions or a single-base mutation

For construction of TN47 (KM22Δ*bvgR*), a 1,520-bp DNA fragment (Eurofins Genomics Blue Heron, Bothell, WA) containing a 759-bp upstream region, which included 755 bps upstream of *bvgR* and the first two bps of *bvgR*, and a 761-bp downstream region, which included the last 4 bp of *bvgR* and extending 751 bps downstream of *bvgR*, flanked by SpeI and BamHI sites was cloned, following digestion, into pSS4245 using the same sites. The resulting plasmid pTN90 was then transformed into *E. coli* MFD*pir,* with selection on LA supplemented with kanamycin and DAP. The recipient KM22 was grown on BG agar supplemented with streptomycin and 50 mM MgSO_4_ prior to mating, which was performed on BG agar supplemented with 10 mM MgCl_2_ and 50 mM MgSO_4_ for 5 h at 37°C. Cointegrants were selected on BG agar supplemented with 50 mM MgSO_4_ and carbenicillin and kanamycin. Colonies arising from this selection were then then plated on BG agar supplemented with streptomycin. Resulting colonies were then screened for the loss of the suicide vector by sensitivity to carbenicillin and by PCR for incorporation of the deletion allele. A single colony, designated TN47 hence forth referred to as Δ*bvgR*, was selected for subsequent use and was further confirmed by PCR amplification of the upstream and downstream regions of the *bvgR* gene followed by DNA sequence analysis.

For the Δ*risA* construction, two 500 bp DNA fragments flanking the region to be deleted, i.e., from the first 5 to the last 5 codons of *risA*, were generated by PCR using KM22 genomic DNA as a template and primer pairs Q2186 (5′-tatggtctccggccgccacgaccgcgtcgtcggccgccgcgc) & Q2230 (5′- tatggtctccaattggttttgcgtgttcatggccggaaatgtaacagtgacaagaaccagc), and Q2231 (5′-tat ggtctccaattggatggcggcagttgacctaatggcccgccccgggcaggagcgc) & Q2203 (5′-tatggtctcggatcccagcgccgtggcgccccagcccagccattcgatgcc). Following *Bsa*I restriction enzyme digestion, the resulting *Not*I-*Mfe*I and *Mfe*I-*BamH*I fragments were ligated together with *Not*I and *BamH*I cleaved pSS4894 to generate plasmid pQC2428 (pSS4894::*ΔrisA*, Gent^R^) containing a *Mfe*I site at the deletion point between the two flanking regions. To facilitate the allelic exchange, a 762-bp DNA fragment (Biomatik USA, Wilmington, DE) encoding a chloramphenicol resistance cassette flanked by *EcoR*I sites was cloned, following digestion, into pQC2428 to create pQC2429 (pSS4894::*ΔrisA*::CM, CM^R^, Gent^R^). Both pQC2428 and pQC2429 were transformed into *E. coli* RHO3, with selection on LA supplemented with gentamicin and DAP, and used as donor strains.

Construction of QC5012 (KM22 *ΔrisA*) was created employing a two-step process. The first step resulted in the construction of QC5010 (KM22 *ΔrisA::CM*), in which the internal portion of the *risA* allele was replaced with a chloramphenicol resistance cassette. To introduce ΔrisA::*CM*, the donor RHO3[pQC2429] was swabbed together with the KM22 recipient onto BG agar supplemented with DAP. After incubation for 3 h at 37°C the cells were streaked onto selective media, i.e., BG plus chloramphenicol and grown for 3 days at 37°C. Allelic replacement under these conditions occurs via a single step involving two homologous recombination events in the flanking DNA of the plasmid that is linearized following conjugative transfer, induction of I-*Sce*I synthesis, and cleavage at the I-*Sce*I site in the vector. A candidate for replacement of *risA* with *ΔrisA::CM* was selected, screened, and saved as strain QC5010 (KM22 *ΔrisA::CM*). To replace *ΔrisA::CM* with an *ΔrisA* in the second step, QC5010 (KM22 *ΔrisA::CM*) was used as a recipient in a mating with RHO3[pQC2428]. This mating was performed maintaining modulation of the recipient KM22 in all steps by the addition of 50 mM MgSO_4_ to the recipient growth plate, the mating plate, and the selection plate. In addition, the mating plate contained DAP, and the selection plate contained chloramphenicol and gentamicin. Plasmid-integrants arising from this mating were subsequently restreaked onto plain BG plate. Under such conditions, synthesis of I-*Sce*I is induced, integrated plasmid is cleaved, and survivors arise by repair of the double-strand break by homologous recombination. Those in which the *ΔrisA* replaced the *ΔrisA::CM* allele were identified by their chloramphenicol-sensitive phenotype. One of these was chosen as QC5012 (KM22 *ΔrisA*) and confirmed by PCR and sequencing.

For the Δ*risS* construct, two 500 bp DNA fragments flanking the region to be deleted, i.e., from the first 5 to the last 5 codons of risS, were generated by PCR using KM22 genomic DNA as a template and primer pairs Q2204(5′ tatggtctccggccgcatctgccgccgcctgcgcggcggccacgac) & Q2209 (5′ tatggtctcaattgctcctgcccggggcgggccattaggtca), and Q2210 (5′ tatggtctcaattgttagacagccaaaaccaatagcgtag) & Q2189(5′ tatggtctccgga tccatcgtgttgtcggggtcgacgatg). Following *Bsa*I cleavge, the resulting *Not*I-*Mfe*I and *Mfe*I-*BamH*I fragments were ligated together with *Not*I and *BamH*I cleaved pSS4894 to generate plasmid pQC2417 (pSS4894::*ΔrisS*, Gent^R^) containing a *Mfe*I site at the deletion point between the two flanking regions. To facilitate the allelic exchange, a 762-bp DNA fragment (Biomatik USA, Wilmington, DE) encoding a chloramphenicol resistance cassette flanked by *EcoR*I sites was cloned, following digestion, into pQC2417 to create pQC2430 (pSS4894::*ΔrisS*::CM, CM^R^, Gent^R^). Both pQC2417 and pQC2430 were transformed into *E. coli* RHO3, with selection on LA supplemented with gentamicin and DAP, and used as donor strains.

Construction of QC5013 (KM22 *ΔrisS*) was created employing a two-step process similarly as described above. The first step resulted in the construction of QC5011 (KM22 *ΔrisS::CM*), using the donor RHO3[pQC2430] and the KM22 recipient. To replace *ΔrisS::CM* with an *ΔrisS* in the second step, QC5011 (KM22 *ΔrisA::CM*) was used as a recipient in a mating with RHO3[pQC2417] to create QC5013 which was confirmed by PCR and sequencing.

For the Δ*risAS* construct, two 500 bp DNA fragments flanking the region to be deleted, i.e., from the first 5 of *risA* to the last 5 codons of *risS*, were generated by PCR using KM22 genomic DNA as a template and primer pairs Q2186 (5′ tatggtctccggccgccacgaccgcgtcgtcggc cgccgcgc) & Q2230 (5′ tatggtctccaattggttttgcgtgttcatggccggaaat gtaacagtgacaagaaccagc), and Q2210 (5′ tatggtctcaattgtt agacagccaaaaccaatagcgtag) & Q2189 (5′T atggtctccggatccatc gtgttgtcggggtcgacgatg). Following *Bsa*I digestion, the resulting *Not*I-*Mfe*I and *Mfe*I-*BamH*I fragments were ligated together with *Not*I and *BamH*I cleaved pSS4894 to generate plasmid pQC2426 (pSS4894::*ΔrisAS*, Gent^R^) containing a *Mfe*I site at the deletion point between the two flanking regions. To facilitate the allelic exchange, a 762-bp DNA fragment (Biomatik USA, Wilmington, DE) encoding a chloramphenicol resistance cassette flanked by *EcoR*I sites was cloned, following digestion, into pQC2426 to create pQC2427 (pSS4894::*ΔrisAS*::CM, CM^R^, Gent^R^). Both pQC2426 and pQC2427 were transformed into *E. coli* RHO3, with selection on LA supplemented with gentamicin and DAP, and used as donor strains.

Construction of QC5014 (KM22 *ΔrisAS*) was created employing a two-step process similarly as described above. The first step resulted in the construction of QC5009 (KM22 *ΔrisAS::CM*), using the donor RHO3[pQC2427] and the KM22 recipient. To replace *ΔrisAS::CM* with an *ΔrisS* in the second step, QC5009 (KM22 *ΔrisAA::CM*) was used as a recipient in a mating with RHO3[pQC2426] to create QC5014 which was confirmed by PCR and sequencing.

To obtain the risAD60N allele (Chen et al., 2017), fragments comprising sequences flanking the mutation were synthesized using PCR amplification with KM22 genomic DNA as a template. The upstream fragment was amplified with primers 3493* (5′-tataggtctccggccgcggtggtgaaggccaccttgtc) and 3499 (5′-tatag gtctccatcaggttgagaaccagcaggtcaaagtg). The downstream fragment was amplified with primers 3501 (5′-tataggtctcctgatgctgccgggcg aggatggcctgtcgatc) and 3496* (5′-tataggtctcggatccgatctggccgag gtcctcgtcgatg). Both fragments were digested with the *Bsa*I to create cohesive ends compatible with *Eag*I at one end of the first PCR fragment and with *BamH*I at one end of the second PCR fragment. *Bsa*I cleavage also generated cohesive ends, allowing two PCR fragments joined in the middle along with the D60N mutations incorporated into the primer 3499. The two *Bsa*I digested fragments were ligated together with pSS4984 digested with *Eag*I and *BamH*I, transformed, and screened to create pSS5083. The resulting plasmid pSS5083 was then transformed into *E. coli* RHO3, with selection on LA supplemented with gentamicin and DAP.

To construct QC5402 (KM22 *risAD60N*), QC5010 (KM22 *ΔrisA::CM*) was used as a recipient in a mating with RHO3[pSS5083]. This mating was performed maintaining modulation of the recipient *KM22* in all steps by the addition of 50 mM MgSO_4_ to the recipient growth plate, the mating plate, and the selection plate. In addition, the mating plate contained DAP, and the selection plate contained chloramphenicol and gentamicin. Plasmid-integrants arising from this mating were subsequently restreaked onto plain BG plate. One of those in which the *risAD60N* replaced the *ΔrisA::CM* allele was identified as QC5402 by its chloramphenicol sensitive, PCR and sequencing.

### Growth kinetics of *Bordetella bronchiseptica* strains

For each strain, a single colony was inoculated in SS broth supplemented with 40 μg/mL streptomycin and grown overnight at 37°C with shaking at 275 rpm. Overnight cultures were then diluted to an OD_600_ of 0.5 in SS broth supplemented with 40 μg/mL streptomycin and with or without 50 mM MgSO_4_. 100 μL of culture for each strain (biological replicates) were transferred into four wells (technical replicates) of a microplate. Plates were incubated in a high throughput microplate reader (Bioscreen C, Growth Curves USA, Piscataway, NJ) at 37°C with gentle agitation and the OD_600_ was measured every 30 min for 24 h. Results were analyzed using Prism 9 Software (GraphPad, San Diego, CA). At least four independent experiments with four technical replicates in each experiment were performed. After 11 h of growth at 37°C with shaking at 275 rpm, serial dilutions of each strain were plated on BG agar plates to determine CFU/mL and to confirm the expected colony morphology and hemolytic phenotype.

### RNA extraction of *Bordetella bronchispetica* strains

For each strain, a single colony was inoculated in 14 mL polystyrene round-bottom tubes (Corning Falcon, Glendale, AZ) containing 2 mLs SS broth supplemented with 40 μg/mL streptomycin and grown overnight at 37°C with shaking at 275 rpm. Overnight cultures were then diluted to an OD_600_ of 0.5 in a 250-ml flask containing 10 mL SS broth supplemented with 40 μg/mL streptomycin and with or without 50 mM MgSO_4_ and incubated for 11 h at 37°C with shaking at 275 rpm. Four biological replicates were prepared for each strain and grown in non-modulating (Bvg+) conditions and in modulating (Bvg-) conditions. For RNA extraction, bacteria were pelleted by centrifugation at 4,000 × g at 4°C for 10 min. Supernatant was decanted, and bacteria were resuspended in TRI reagent (ThermoFisher Scientific, Waltham, MA), incubated at room temperature for 5 min, then stored at-80°C until RNA isolation. RNA was extracted from samples using the Direct-zol RNA Miniprep Kit (Zymo Research, Irvine, CA) following manufacturer’s instructions. On-column DNase treatment was performed using the RNase-free DNase set (Qiagen, Germantown, MD). RNA samples were then further processed using an RNA Clean & Concentrator kit (Zymo Research, Irvine, CA) following manufacturer’s instructions. To remove residual prokaryotic rRNA, the DNA-free total RNA samples were treated with the Ribo-Zero Gold Epidemiology Kit (Illumina, San Diego, CA) according to manufacturer’s instructions. Removal of rRNA was assessed using the Agilent 2,100 Bioanalyzer RNA 6000 Pico kit (Agilent Technologies, Santa Clara, CA). Samples were submitted to the Iowa State University DNA Facility in Ames, IA for library preparation using the Stranded Total RNASeq library preparation kit (Illumina, San Diego, CA). Libraries were sequenced on two flow cells using the Illumina HiSeq 3,000 platform to generate 150-nucleotide single-end reads on high output mode.

### Gene expression analysis

The expression analysis approach consisted of multiple open-source software. Sample read quality was checked using FastQC (Blankenberg et al., 2010) prior to alignment which was performed using BWA-MEM (Li and Durbin, 2010).^1^ Unnormalized (raw) read counts and QC based on a minimum quality score of 20 and only unique mapping reads were performed using FeatureCounts with final expression values being determined using DeSeq2 (Liao et al., 2014; Love et al., 2014). Data was organized by mutant vs. wildtype modulated, mutant vs. wildtype non-modulated, and non-modulated vs. modulated. An FDR cut-off of ≤0.1 was used to filter the expression data based on a KM22 GenBank assembly file (accession number CP022962.2) to create a GFF formatted file for annotation. The data was based on the reverse-strand counts and genes showing an FDR of 0.00E+00 were included as this value is inserted by the software due to the floating point for *p*-values in R having a max value of ~1E-308.

### Expression visualization and analysis

The gene list along with the annotations from RAST and the KM22 locus tags were used to create GCT 1.2 formatted files for heatmap visualization and hierarchical clustering analysis. The GCT file is a tab-separated text file. The file has the GCT version on the first row, the “# of rows of data,” and the “# of the number of columns” with numerical data on the second row. The third row starts the data columns, with the first column being row names (ex. gene Id’s) and the expression values for each group (header) in subsequent columns. The number of columns must match the value on the second row of the GCT 1.2 file. The GCT file was then used as input for the expression matrix visualization program MORPHEUS.^2^ Within MORPHEUS, hierarchical clustering of the expression values was carried out using a distance method metric of 1-Pearson correlation and complete linkage. Clustering was performed on columns and rows. For heatmaps, all duplicate gene names and all genes with an “NA” value were removed. All hypothetical and putative KM22 locus genes were also removed and annotation based on KM22 locus tags using MG-RAST were added (Keegan et al., 2016). Filtering of these ambiguous tags reduced the gene list from 4,807 KM22 locus tags to 2,337 unique gene descriptions from RAST.

### Statistical visualization

Treatment and control group samples were compared across expression values to examine the presence or lack of collinearity among the samples. Statistical analysis of collinearity was performed using the Variables exploration package in Galaxy.^3^ A correlation matrix was obtained and visualized as a PCA plot (Supplementary Figure S2) using R packages ggplot2 and ggfortify (Wickham, 2009; Tang et al., 2016). The PCA plot demonstrated that the different groups clustered together discretely based on the treatment classes used in each comparison.

### cDNA preparation and qPCR

rRNA depleted and DNA-free RNA (1 μg 150 ng per 20 μL RT reaction) from each biological replicate was reverse transcribed using 300 ng of random oligonucleotide hexamers and LunaScript RT SuperMix Kit (New England Biolabs, Ipswich, MA) according to the manufacturer’s protocol. The resulting cDNA was diluted 1:1,000, and 1 μL of this dilution was used in qPCR reactions containing 250 nM primers and Luna Universal qPCR Master Mix (New England Biolabs, Ipswich, MA) using a QuantStudio 3 real-time PCR detection system (Thermo Fisher Scientific, Waltham, MA). Primers were designed using Geneious Prime (Biomatters Ltd., Auckland, New Zealand) and listed in Supplementary Table S1. To confirm the lack of DNA contamination, reactions without reverse transcriptase were performed. Dissociation curve analysis was performed for verification of product homogeneity. Threshold fluorescence was established within the geometric phase of exponential amplification and the Ct value for each reaction was determined. Fold-change in transcript level was determined using the relative quantitative method (ΔΔCT) (Livak and Schmittgen, 2001) with the *recA* amplicon used as the standardization control.

### Extraction of c-di-GMP and GMP

Nucleotide extractions for all *B. bronchiseptica* strains were performed as previously described with slight modifications (Monds et al., 2010; Newell et al., 2011; Sisti et al., 2013; Jia et al., 2014). Briefly, a single colony from each strain was inoculated in SS broth supplemented with 40 μg/mL streptomycin and grown overnight at 37°C with shaking at 275 rpm. Overnight cultures were then diluted to an OD_600_ of 0.025 in 20 mLs SS broth supplemented with 40 μg/mL streptomycin and incubated for 24 h at 37°C with shaking at 275 rpm. Bacteria were pelleted by centrifugation at 10,000 × g at 4°C for 5 min. Prior to centrifugation, 100 μL of culture was removed for serial dilutions in PBS and then plated on blood agar plates to determine colony forming units (CFU)/mL counts. After centrifugation, supernatant was decanted, and bacteria were resuspended in cold 5 mL extraction buffer [methanol:acetonitrile:water (40:40:20) plus 0.1 M formic acid at −20°C], and 25 μL of 25 nM of 8-Bromoguanosine 3′,5′-cyclic monophosphate sodium salt (8-Br-cGMP/internal standard) (Millipore Sigma, Burlington, MA) was added. Samples were then incubated for 30 min at −20°C. Insoluble material was then removed by centrifugation at 20,000 × g at 4°C for 10 min and the supernatant containing the nucleotide extract was saved. The extraction procedure was repeated two more times on the retained cell pellet. Combined supernatants were then incubated overnight in SpeedVac using low heat and the residue containing extracted nucleotides was resuspended in 100 μL of water and stored at-80°C until quantified using liquid chromatography–tandem mass spectrometry (LC–MS/MS) analysis. Stock solutions of c-di-GMP (Biolog Life Science, Bremen, Germany) and GMP (Cayman Chemical, Ann Arbor, MI) were prepared and diluted for use as standards. 25 nM of 8-Br-cGMP was added to all standards. A minimum of four extractions were performed for each strain and final c-di-GMP and GMP concentrations were expressed as nmol/1×10^9^ CFU. Results were analyzed for significance using Prism 9 Software (GraphPad, San Diego, CA). Means were compared using a one-way analysis of variance (ANOVA) with a Dunnett’s multiple comparison post-test. A 5% level of significance (*p* < 0.05) was considered significant.

### Quantification of c-di-GMP by LC–MS/MS

Nucleotide extracts were submitted to the W.M. Keck Metabolomics Research Laboratory (Office of Biotechnology, Iowa State University, Ames IA). LC separations were performed with an Agilent Technologies 1,290 Infinity II UHPLC instrument coupled to a 6,470 triple quadrupole mass spectrometer with an electrospray ionization (ESI) source (Agilent Technologies, Santa Clara, CA). All solvents were LC–MS grade and were obtained from Fisher Chemical (Thermo Fisher Scientific Waltham, MA). Running solvents consisted of A: 0.1% formic acid in water with 1% methanol and B: 0.1% formic acid in 70% acetonitrile with 25% methanol and 5% water. The flow rate was 0.75 mL/min starting at 0% B and time 0 which was increased on a gradient to 25% B at 8 min, which was maintained until 12 min before returning to 0% B at 15 min runtime, a 5-min post run equilibration was preformed after each sample injection. The mass spectrometer was equipped with an electrospray ion source and was operated in positive and mode. Target molecules were detected using electrospray ionization in positive ionization mode. Nitrogen was used as the service gas for the ion source with a drying gas flow rate of 12 L/min at a temperature of 350°C, a nebulizing pressure of 40 psi, and a sheath gas flow of 11 L/min at 400°C. The capillary, nozzle, and fragmentor voltages were 4,000, 0, and 135 volts, respectively. The mass spectrometer was operated in multiple reaction monitoring (MRM) mode with MS/MS transitions targeting selected molecules (Table 2).

**Table 2:** LC–MS/MS parameters for the measured transitions of cyclic nucleotide monophosphates (cNMPs) and internal standard (IS).

Name	Type	Precursor ion	Product ion	Retention time	Ion polarity	Collision energy
c-di-GMP	Target (cNMP)	691.1	152.1	6.048	Positive	25
GMP	Target (cNMP)	364.1	152.1	5.699	Positive	25
2′,3’-Br-cGMP	8-Br-cAMP (IS)	424	230	7.680	Positive	25

LC–MS/MS analysis was based on previously published methods (Jia et al., 2014). Fifty microliter of liquid chromatography (LC) running solvent B was added to 100 μL of each extract and filtered with 0.2-micron centrifugal filters (Millipore Sigma, Burlington, MA) immediately prior to LC–MS/MS analysis. The samples were maintained in the autosampler chamber at 10°C. A volume of 20 μL of each sample was injected into the LC system and separated on a SphereClone 5 μm ODS(2) 80 A, LC Column 250 × 4.6 mm (Phenomenex, Torrance, CA), at 25°C, and an electrospray ion source operated in positive mode. LC–MS/MS peak quantification was accomplished using Agilent MassHunter Quantitative Analysis (version 10.0) software (Agilent Technologies, Santa Clara, CA) with all target analyte reported amounts calculated relative to the standard curve for each analyte and to the internal standard (2′,3’-Br-cGMP) for each sample.

### Soft agar swimming motility assay

Motility assays were performed as previously described (Nicholson et al., 2012a; Sukumar et al., 2014). Briefly, plates were prepared with Stainer-Scholte medium (SSM) containing 0.35% agarose, supplemented with or without 50 mM MgSO_4_ and marked with a cross to divide each plate into four quadrants. For each strain, a single colony was stab inoculated into each quadrant of a SSM plate and an SSM + MgSO_4_ plate and incubated at 37°C for 48 h. Motile organisms display outward migration from the point of inoculation and the diameter of the migration zone was measured at 24 and 48 h. Four experiments were performed. Results were analyzed for significance using Prism 9 Software (GraphPad, San Diego, CA). Means were compared using a two-way analysis of variance (ANOVA) with a Dunnett’s multiple comparisons test to compare differences among strains for each time point. A 5% level of significance (*p* < 0.05) was considered significant.

### Microtiter plate assay for static biofilm formation

A static biofilm assay was performed using crystal violet was performed as previously described (Nicholson et al., 2012b; Sukumar et al., 2014; Waack and Nicholson, 2018). Briefly, a single colony for each strain was inoculated in SS broth supplemented with 40 μg/mL streptomycin and grown overnight at 37°C with shaking at 275 rpm. Overnight cultures were then diluted to an OD_600_ of 0.250 in SS broth supplemented with 40 μg/mL streptomycin. Hundred microliter of culture for each strain (biological replicates) was transferred into 10 wells (technical replicates) of a U bottom, non-tissue-culture treated, 96-well polyvinyl chloride microtiter plate (BD Falcon, Corning, Kennebunk, ME). Plates were incubated statically for 24 h in a humidified 37° C incubator. The cultures were aspirated from the plate wells and each well was washed 3 times with 200 μL sterile PBS. Biofilms were fixed by the addition of 200 μL 100% ethanol and dried for 10 min. Biofilms were stained by the addition of 200 μL 0.1% crystal violet (CV) (Sigma, St. Louis, MO) to each well and incubated for 15 min. CV was removed and the wells were washed 3 times with 200 μL sterile PBS and the plate allowed to dry overnight. Bound CV dye was eluted by incubation for 10 min with 200 μL 100% ethanol. One hundred and twenty five microliter of the eluted CV was transferred to a new 96-well plate and biofilm biomass was quantified by measuring the absorbance at 595 nm (A595) in a microplate reader (SpectraMax M5, Molecular Devices, Sunnyvale, CA). Six independent experiments were performed. Results were analyzed for significance using Prism 9 Software (GraphPad, San Diego, CA). Means were compared using a one-way analysis of variance (ANOVA) with a Dunnett’s multiple comparison post-test. A 5% level of significance (*p* < 0.05) was considered significant.

### Data availability statement

All raw sequencing reads have been deposited in the Sequence Read Archive (SRA) at under the BioProject accession number PRJNA1018256.

## Results

To characterize the transcriptional responses governed by BvgR, RisA, and RisS, mutants harboring an in-frame deletion in each of the genes encoding these proteins were constructed in KM22. Additionally, an aspartate to asparagine mutation at D60 (D60N) in RisA was constructed to abolish the phosphorylated form of RisA, allowing the role of phosphorylation in transcriptional regulation to be evaluated (Chen et al., 2017). Growth of each mutant, along with KM22, was monitored for 24 h in non-modulating (Bvg+) conditions (Supplementary Figure S1A) or in modulating (Bvg-) conditions with 50 mM MgSO_4_ (Supplementary Figure S1B). No differences in growth kinetics were observed among strains grown in the same culture condition (Supplementary Figures S1A,B), indicating that each mutation did not produce a defect in growth rate. Additionally, 11 h was chosen as the optimal time-point to collect bacteria for RNA extraction because all isolates were in the exponential stage of growth (Supplementary Figures S1A,B). Similar CFU levels were then recovered from KM22 (WT) and all mutants after 11 h of growth in non-modulating (Bvg+) conditions and similar, yet higher CFU levels, were recovered from all strains grown in modulating (Bvg-) conditions (Supplementary Figure S1C). RNA-seq analyses were performed on KM22 (WT) and all mutants grown in non-modulating (Bvg+) conditions or grown in modulating (Bvg-) conditions to fully delineate the differential expression of each mutant in both conditions. A PCA plot based on correlation matrix of expression values for all genes in the experiment shows that the samples were positively correlated within the conditions they were treated (Supplementary Figure S2). The comparison of WT grown in non-modulating (Bvg+) conditions versus modulating (Bvg-) conditions was used to establish the full spectrum of vags and vrgs and included for reference in all comparisons for each mutant evaluated. In accordance with tradition, genes more abundant or upregulated in non-modulating (Bvg+) conditions were considered vags. Conversely, genes less abundant or downregulated in non-modulating (Bvg+) conditions were considered vrgs.

### BvgR in non-modulating (Bvg+) conditions

**Figure 1:**
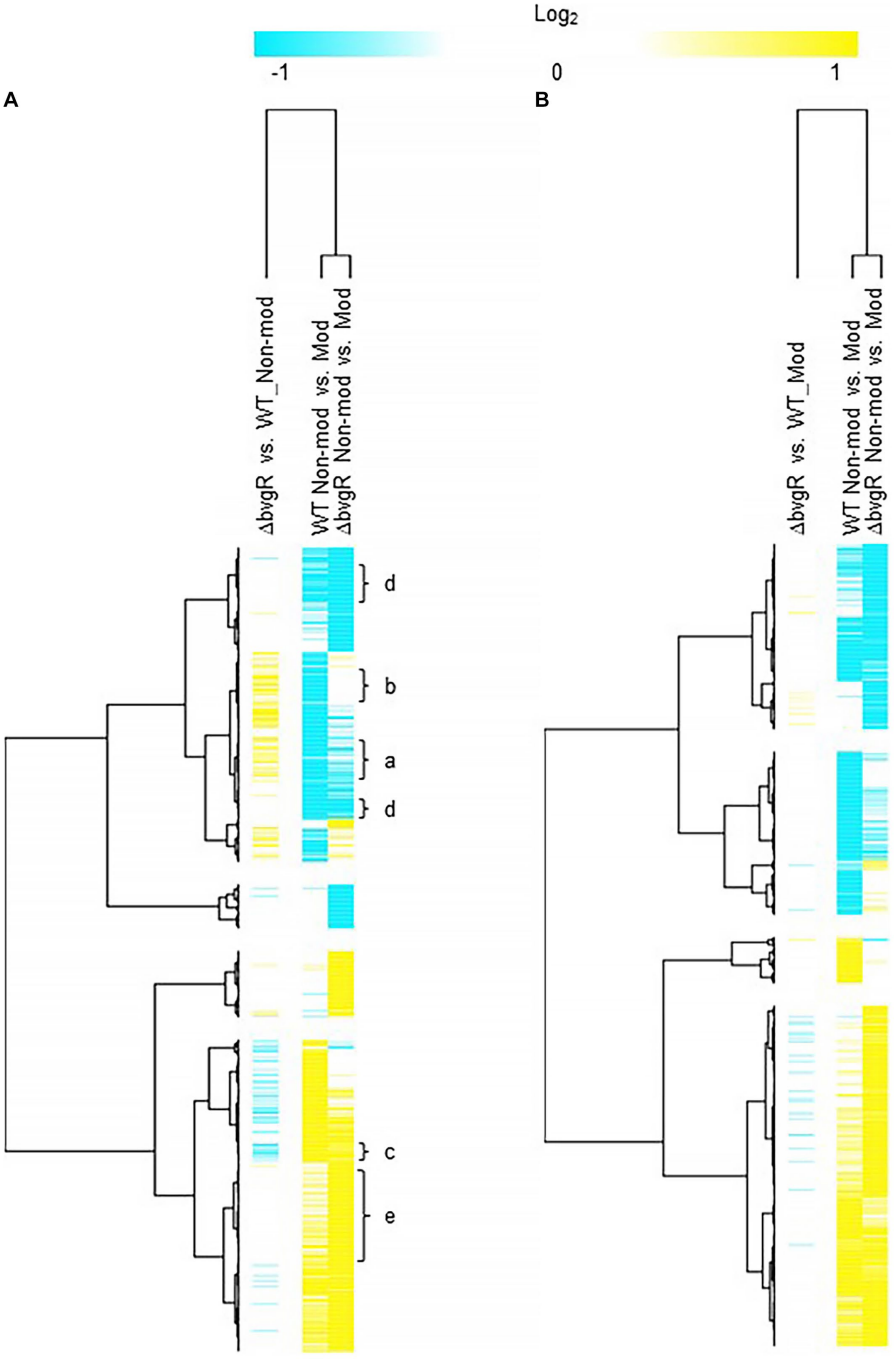
Hierarchical cluster heatmap displaying the transcriptional response of *ΔbvgR* in non-modulating (Bvg+) conditions and modulating (Bvg-) conditions. **(A)** Hierarchical cluster heatmap displaying the transcriptional response of *ΔbvgR* in non-modulating (Bvg+) conditions. **(B)** Hierarchical cluster heatmap displaying the transcriptional response of *ΔbvgR* in modulating (Bvg-) conditions. Hierarchical clustering of the expression values was carried out using a distance method metric of 1-Pearson correlation and complete linkage. Clustering was performed on columns and rows. Dendrograms are on the left side and on top of the heat map. Isolates names and specific comparisons are provided at the top of the heat map. All expression profiles of genes are in row and are represented using the color scale at top. Yellow, indicates increased gene expression; blue, decreased gene expression; white, no significant change in gene expression. Letters represent clusters of genes with similar transcription pattern.

When *ΔbvgR* was compared to WT in non-modulating (Bvg+) conditions, 126 genes were upregulated in *ΔbvgR* and 61 genes were downregulated (Supplementary Table S1). Of the genes upregulated in *ΔbvgR*, 98 were vrgs and include capsule genes and chemotaxis/ motility genes [Figure 1A (clusters a and b); Supplementary Table S1 (clusters a and b)]. The transcriptional profile of these genes indicated that they are repressed by BvgR in non-modulating (Bvg+) conditions. These genes can be further divided into vrgs that either maintained (cluster a) or lost (cluster b) an expected vrg transcriptional response, i.e., repressed or downregulated in non-modulating (Bvg+) conditions. Vrgs upregulated in *ΔbvgR* and maintained a classical vrg transcriptional response when *ΔbvgR* grown in non-modulating (Bvg+) conditions was compared to modulating (Bvg-) conditions include *flaA* and other chemotaxis/ motility genes [Figure 1A (cluster a); Table 3; Supplementary Table S1 (cluster a)]. The transcriptional profile of these genes indicated that although they were repressed by BvgR in non-modulating (Bvg+) conditions, BvgR is not required for modulation. In contrast, no significant change in gene expression was observed for 61 vrgs when *ΔbvgR* grown in non-modulating (Bvg+) conditions was compared to modulating (Bvg-) conditions. The no significant change in gene expression observed between non-modulating (Bvg+) and modulating (Bvg-) conditions by these vrgs indicated that they lost the expected vrg transcriptional response of repression or downregulation during non-modulating (Bvg+) conditions in the absence of BvgR. Genes within this cluster include *wcbA* and other capsule genes [Figure 1A (cluster b); Table 3; Supplementary Table S1 (cluster b)]. The transcriptional profile of these genes indicated that they were repressed by BvgR in non-modulating (Bvg+) conditions and BvgR was required for modulation.

**Table 3:** ΔbvgR in non-modulating (Bvg+) conditions.

Cluster	ΔbvgR vs. WT Non-Mod	WT Non_Mod vs. Mod	ΔbvgR Non-mod vs. Mod	Description	Gene
a	Up	Down	Down	Vrgs repressed by BvgR in non-modulating (Bvg+) conditions and do not require BvgR for modulation	*flaA* (flagellar protein)
b	Up	Down	NS	Vrgs repressed by BvgR in non-modulating (Bvg+) conditions and require BvgR for modulation.	*wcbA* (capsular polysaccharide)
c	Down	Up	Up	Vags that require BvgR for full expression in non-modulating (Bvg+) conditions	PRN
d	NS	Down	Down	Vrgs not regulated by BvgR in non-modulating (Bvg+) conditions	*ureE* (urease)
e	NS	Up	Up	Vags not regulated by BvgR in non-modulating (Bvg+) conditions	FHA

Transcriptional response of genes within clusters identified from comparisons used to evaluate the differential expression of the ΔbvgR mutant in non-modulating (Bvg+) conditions. Yellow, indicates genes upregulated (Up) (log2fc > 1); blue, indicates genes downregulated (Down) (log2fc < −1); white, no significant change or NS in gene expression (log2fc < 1, but > −1).

Thirty-four vags were downregulated in *ΔbvgR* when *ΔbvgR* was compared to WT in non-modulating (Bvg+) conditions and maintained a classical vag transcriptional response when *ΔbvgR* grown in non-modulating (Bvg+) conditions was compared to modulating (Bvg-) conditions, i.e., they were upregulated in non-modulating (Bvg+) conditions. Genes that exhibited this transcriptional response include known virulence factors, such as PRN [Figure 1A (cluster c); Table 3; Supplementary Table S1 (cluster c)]. The transcriptional profile of these genes indicated that BvgR is required for full expression in non-modulating (Bvg+) conditions.

Focusing on vrgs, 314 vrgs showed no significant change in gene expression between *ΔbvgR* and WT in non-modulating (Bvg+) conditions, indicating that these genes were not repressed by BvgR. One hundred and fifty eight of these vrgs were also downregulated in non-modulating (Bvg+) conditions and therefore maintained a classical vrg transcriptional response when *ΔbvgR* grown in non-modulating (Bvg+) conditions was compared to modulating (Bvg-) conditions. Genes that exhibited this transcriptional response include nutrient transport and metabolism genes, such as *ureE* [Figure 1A (cluster d); Table 3; Supplementary Table S1 (cluster d)]. The transcriptional profile of these genes indicated that they are not regulated by BvgR in non-modulating (Bvg+) conditions.

A total of 227 vags showed no significant change in gene expression between *ΔbvgR* and WT in non-modulating (Bvg+) conditions and maintained an expected vag transcriptional response when *ΔbvgR* grown in non-modulating (Bvg+) conditions was compared to modulating (Bvg-) conditions. Genes that exhibited this transcriptional response include most virulence factors, such as FHA and T3SS genes [Figure 1A (cluster e); Table 3; Supplementary Table S1 (cluster e)]. The transcriptional profile of these genes indicated that they are not regulated by BvgR.

### BvgR in modulating (Bvg-) conditions

Only a few genes were differentially regulated when *ΔbvgR* was compared to WT in modulating (Bvg-) conditions (Figure 1B; Supplementary Table S2). This result agrees with previous findings demonstrating that BvgR is a vag and functions as a repressor to vrgs during non-modulating (Bvg+) conditions.

### RisA and phosphorylation of RisA in non-modulating (Bvg+) conditions

**Figure 2:**
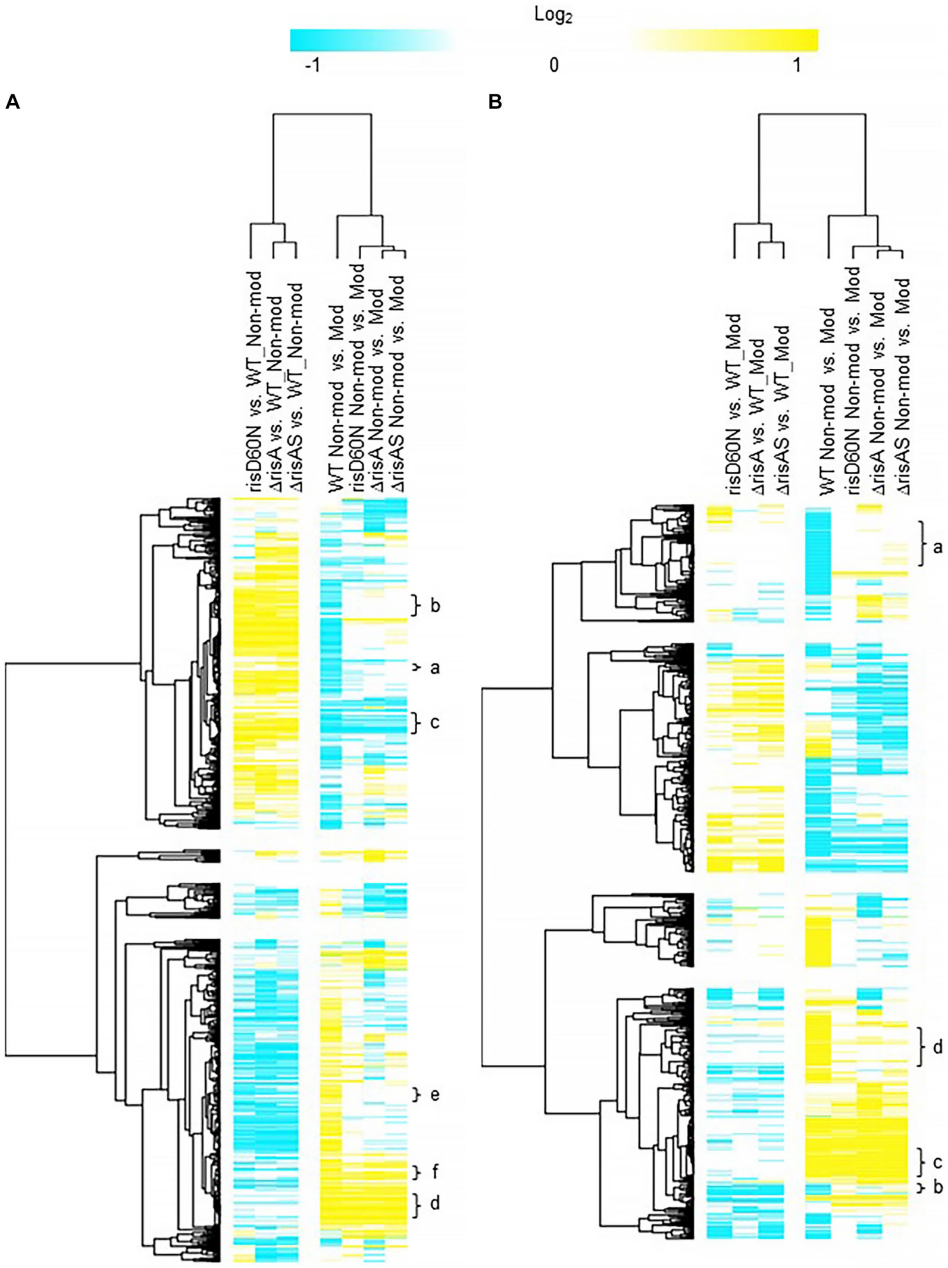
Hierarchical cluster heatmap displaying the transcriptional response of *ΔrisA* in non-modulating (Bvg+) conditions and modulating (Bvg-) conditions. **(A)** Hierarchical cluster heatmap displaying the transcriptional response of *ΔrisA* in non-modulating (Bvg+) conditions. **(B)** Hierarchical cluster heatmap displaying the transcriptional response of *ΔrisA* in modulating (Bvg-) conditions. Hierarchical clustering of the expression values was carried out using a distance method metric of 1-Pearson correlation and complete linkage. Clustering was performed on columns and rows. Dendrograms are on the left side and on top of the heat map. Isolates names and specific comparisons are provided at the top of the heat map. All expression profiles of genes are in row and are represented using the color scale at top. Yellow, indicates increased gene expression; blue, decreased gene expression; white, no significant change in gene expression. Letters represent clusters of genes with similar transcription pattern.

Comparison of *ΔrisA* and *ΔrisAS* to WT in non-modulating (Bvg+) conditions resulted in 321 genes downregulated in *ΔrisA* and *ΔrisAS* compared to WT (Figure 2A; Supplementary Table S3). Of the downregulated genes, 232 were also downregulated in risAD60N compared to WT, indicating that the regulation of these genes is dependent on phosphorylation of RisA. In contrast, 83 genes that were downregulated in *ΔrisA* and *ΔrisAS* compared to WT exhibited no significant change in gene expression in risAD60N compared to WT, indicating that regulation of these genes is not dependent on phosphorylation of RisA (Figure 2A; Supplementary Table S3).

A total of 307 genes were upregulated in *ΔrisA* and in *ΔrisAS* compared to WT in non-modulating (Bvg+) conditions. Of these, 233 were also upregulated in *risAD60N* compared to WT, indicating that the regulation of these genes is dependent of phosphorylation of RisA, while 73 genes exhibited no significant change in gene expression in risAD60N and in WT, suggesting that regulation of these genes is not dependent on phosphorylation of RisA (Figure 2A; Supplementary Table S3).

Focusing on vrgs, 67 exhibited no significant change in gene expression in *ΔrisA*, *ΔrisAS*, and *risAD60N* when compared to WT in non-modulating (Bvg+) conditions and lost an expected vrg transcriptional response, i.e., they were not repressed or downregulated in non-modulating (Bvg+) conditions when growth in non-modulating (Bvg+) conditions was compared to modulating (Bvg-) conditions for each of these mutants. Genes that exhibited this transcriptional response include capsule genes and T6SS genes [Figure 2A (cluster a); Table 4; Supplementary Table S3 (cluster 3)]. The transcriptional response of these genes indicated that expression of these genes in non-modulating (Bvg+) conditions is not dependent on RisA, however RisA and phosphorylation of RisA is required for maximal expression in modulating (Bvg-) conditions.

**Table 4:** *ΔrisA* in non-modulating (Bvg+) conditions.

Cluster	risAD60N vs. WT Non-Mod	ΔrisA vs. WT Non-Mod	ΔrisAS vs. WT Non-Mod	WT Non-Mod vs. Mod	risAD60N Non-Mod vs. Mod	ΔrisA Non-Mod vs. Mod	ΔrisAS Non-Mod vs. Mod	Description	Gene
a	NS	NS	NS	Down	NS	NS	NS	Vrgs not regulated by RisAS and/or phosphorylation of RisA during non-modulating (Bvg+) conditions and require RisAS and phosphorylation of RisA for modulation	*wcbA* (capsular polysaccharide)
b	Up	Up	Up	Down	NS	NS	NS	Vrgs repressed by RisAS and phosphorylation of RisA during non-modulating (Bvg+) conditions and require RisAS and phosphorylation of RisA for modulation	*flaA* (flagellar protein)
c	Up	Up	Up	Down	Down	Down	Down	Vrgs repressed by RisA and phosphorylation of RisAS during non-modulating (Bvg+) conditions but do not require RisAS and phosphorylation of RisA for modulation	*livK* (leucine transport)
d	NS	NS	NS	Up	Up	Up	Up	Vags not regulated by RisAS and/or phosphorylation of RisA during non-modulating (Bvg+) conditions and require RisAS and phosphorylation of RisA for maintaining an expected vag transcriptional response	FHA and PRN
e	Down	Down	Down	Up	NS	NS	NS	Vags that require RisAS and phosphorylation of RisA for full expression in non-modulating (Bvg+) conditions and for maintaining an expected vag transcriptional response	*osmC*
f	Down	Down	Down	Up	Up	Up	Up	Vags that require RisAS and phosphorylation of RisA for full expression in non-modulating (Bvg+) conditions but do not require RisA and phosphorylation of RisA Maxbites01!	*bopN* (T3SS)

Transcriptional response of genes within clusters identified from comparisons used to evaluate the differential expression of the Δ*risA* mutant in non-modulating (Bvg+) conditions. Yellow, indicates genes upregulated (Up) (log2fc > 1); blue, indicates genes downregulated (Down) (log2fc < −1); white, no significant change or NS in gene expression (log2fc < 1, but > −1).

A total of 178 vrgs were upregulated in *ΔrisA* and *ΔrisAS* compared to WT in non-modulating (Bvg+) conditions. Of these, 162 were upregulated in *risAD60N* compared to WT, indicating that the regulation of these genes is dependent on phosphorylation of RisA, while 16 genes exhibited no significant change in gene expression in risAD60N and WT, suggesting that regulation of these genes is not dependent on RisA phosphorylation (Figure 2A; Supplementary Table S3). Genes among the 162 vrgs identified as dependent on RisA phosphorylation include chemotaxis/ motility genes and four genes that contain predicted domains related to cyclic di-GMP metabolism domains [locus tags: CJ015_09185 (EAL domain), CJ015_16540 (HD-GYP domain), CJ015_16560 (PilZ, PilZN domain), *lapD* CJ015_18520 (EAL, GGDEF domain)] (Supplementary Table S3). One hundred and eleven of these vrgs lost an expected vrg transcriptional response, i.e., they were not repressed or downregulated in non-modulating (Bvg+) conditions when growth in non-modulating (Bvg+) conditions was compared to modulating (Bvg-) conditions for each of these mutants [Figure 2A (cluster b); Table 4; Supplementary Table S3 (cluster b)]. The transcriptional response of these genes indicates that they were repressed by RisAS and phosphorylation of RisA during non-modulating (Bvg+) conditions and required RisAS and phosphorylation of RisA for modulation. Genes that exhibited this transcriptional response include *flaA* and other chemotaxis/ motility genes [Figure 2A (cluster b); Table 4; Supplementary Table S3 (cluster b)]. In contrast, 21 vrgs were downregulated in non-modulating (Bvg+) conditions and therefore maintained a classical vrg transcriptional response when growth in non-modulating (Bvg+) conditions was compared to modulating (Bvg-) conditions for each of these mutants [Figure 2A (cluster c); Table 4; Supplementary Table S3 (cluster c)]. The transcriptional profile of these genes suggests that while they are repressed by RisA and phosphorylation of RisAS during non-modulating (Bvg+) conditions, they do not require RisAS and phosphorylation of RisA for modulation.

Eighty two vags exhibited no significant change in gene expression in *ΔrisA*, *ΔrisAS*, and *risAD60N* compared to WT in non-modulating (Bvg+) conditions and were upregulated in non-modulating (Bvg+) conditions and therefore maintained an expected vag transcriptional response when growth in non-modulating (Bvg+) conditions was compared to modulating (Bvg-) conditions for each of these mutants [Figure 2A (cluster d); Table 4; Supplementary Table S3 (cluster d)]. The transcriptional profile of these genes indicated that they are not regulated by RisAS and/or phosphorylation of RisA during non-modulating (Bvg+) conditions and they do not require RisAS and phosphorylation of RisA for modulation. Genes that exhibited this transcriptional response include virulence factors, such as PRN and FHA [Figure 2A (cluster d); Table 4; Supplementary Table S3 (cluster d)].

Seventy six vags were downregulated in *ΔrisA*, *ΔrisAS*, and *risAD60N* when compared to WT in non-modulating (Bvg+) conditions and lost an expected vag transcriptional response, i.e., they were not upregulated in non-modulating (Bvg+) conditions when growth in non-modulating (Bvg+) conditions was compared to modulating (Bvg-) conditions for each of these mutants [Figure 2A (cluster e); Table 4; Supplementary Table S3 (cluster e)]. Genes that exhibited this transcriptional response include *osmC* [Figure 2A (cluster e); Table 4; Supplementary Table S3 (cluster e)]. The transcriptional response of these genes indicated that RisAS and phosphorylation of RisA is required for full expression in non-modulating (Bvg+) conditions and for maintaining an expected vag transcriptional response.

Eight vags, including five T3SS genes, were downregulated in *ΔrisA*, *ΔrisAS*, and *risAD60N* compared to WT in non-modulating (Bvg+) conditions and were upregulated in non-modulating (Bvg+) conditions and therefore maintained a classical vag transcriptional response when growth in non-modulating (Bvg+) conditions was compared to modulating (Bvg-) conditions for each of these mutants [Figure 2A (cluster f); Table 4; Supplementary Table S3 (cluster f)]. The transcriptional response of these genes indicated that the expression of these genes is dependent on RisAS and phosphorylation of RisA in non-modulating (Bvg+) conditions, however, they do not require RisA and phosphorylation of RisA for maintaining an expected vag transcriptional response.

### RisA and phosphorylation of RisA in modulating (Bvg-) conditions

When *ΔrisA* and *ΔrisAS* was compared to WT in modulating (Bvg-) conditions, 75 genes were downregulated in *ΔrisA* and *ΔrisAS*. Of these, 53 were also downregulated in *risAD60N* compared to WT, indicating that the regulation of these genes is dependent on RisA phosphorylation, while 22 genes were equally expressed in *risAD60N* and in WT, indicating that regulation of these genes is not dependent on RisA phosphorylation (Figure 2B; Supplementary Table S4). A total of 102 genes were upregulated in *ΔrisA* and in *ΔrisAS* compared to WT in modulating (Bvg-) conditions. Of these, 47 were upregulated in risAD60N compared to WT, indicating that the regulation of these genes is dependent on RisA phosphorylation, while 55 genes were equally expressed in risAD60N and in WT, suggesting that regulation of these genes is not dependent on RisA phosphorylation (Figure 2B; Supplementary Table S4).

Focusing on vrgs, 174 exhibited no significant change in gene expression in *ΔrisA*, *ΔrisAS*, and *risAD60N* compared to WT in modulating (Bvg-) conditions and lost a vrg transcriptional response, i.e., they were not repressed or downregulated in non-modulating (Bvg+) conditions when growth in non-modulating (Bvg+) conditions was compared to modulating (Bvg-) conditions for each of these mutants [Figure 2B (cluster a); Table 5; Supplementary Table S4 (cluster a)]. Genes that exhibited this transcriptional response include *flaA* and other chemotaxis/ motility genes [Figure 2B (cluster a); Table 5; Supplementary Table S4 (cluster a)]. The transcriptional response of these genes indicated that while they are not regulated by RisAS and phosphorylation of RisA in modulating (Bvg-) conditions, RisAS and phosphorylation of RisA was required for modulation.

**Table 5:** *ΔrisA* in modulating (Bvg-) conditions.

Cluster	risAD60N vs. WT Mod	ΔrisA vs. WT Mod	ΔrisAS vs. WT Mod	WT Non-Mod vs. Mod	risAD60N Non-Mod vs. Mod	ΔrisA Non-Mod vs. Mod	ΔrisAS Non-Mod vs. Mod	Description	Gene
a	NS	NS	NS	Down	NS	NS	NS	Vrgs not regulated by RisAS and phosphorylation of RisA in modulating (Bvg-) conditions and require RisAS and phosphorylation of RisA for modulation.	*flaA* (flagellar protein)
b	Down	Down	Down	Down	NS	NS	NS	Vrgs that require RisA and phosphorylation of RisA for full expression in modulating (Bvg-) conditions and require RisA and phosphorylation of RisA for modulation.	*wza* (Capsular polysaccharide)
c	NS	NS	NS	UP	UP	UP	UP	Vags not regulated by RisAS and phosphorylation of RisA during modulating (Bvg-) conditions and do not require RisAS and phosphorylation of RisA for maintaining an expected vag transcriptional response	ACT
d	NS	NS	NS	UP	NS	NS	NS	Vags not regulated by RisAS and phosphorylation of RisA during modulating (Bvg-) conditions but require RisAS and phosphorylation of RisA for maintaining an expected vag transcriptional response.	*bdcB* (GGDEF domain)

Transcriptional response of genes within clusters identified from comparisons used to evaluate the differential expression of the Δ*risA* mutant in non-modulating (Bvg-) conditions. Yellow, indicates genes upregulated (Up) (log2fc > 1); blue, indicates genes downregulated (Down) (log2fc < −1); white, no significant change or NS in gene expression (log2fc < 1, but > −1).

A group of 11 vrgs were downregulated in *ΔrisA*, *ΔrisAS*, and *risAD60N* compared to WT in modulating (Bvg-) conditions and lost an expected vrg transcriptional response, i.e., they were not repressed or downregulated in non-modulating (Bvg+) when growth in non-modulating (Bvg+) conditions was compared to modulating (Bvg-) conditions for each of these mutants [Figure 2B (cluster b); Table 5; Supplementary Table S4 (cluster b)]. Genes that exhibited this transcriptional response include capsule and T6SS locus [Figure 2B (cluster b); Table 5; Supplementary Table S4 (cluster b)]. The transcriptional response of these genes indicated that they require RisA and phosphorylation of RisA for full expression in in modulating (Bvg-) conditions and require RisA and phosphorylation of RisA for modulation.

When evaluating expression profiles of vags, 88 exhibited no significant change in gene expression in *ΔrisA*, *ΔrisAS*, and *risAD60N* compared to WT in modulating (Bvg-) conditions and were also upregulated in non-modulating (Bvg+) conditions and therefore maintained an expected vag transcriptional response when growth in non-modulating (Bvg+) conditions was compared to modulating (Bvg-) conditions for each of these mutants [Figure 2B (cluster c); Table 5; Supplementary Table S4 (cluster c)]. Many genes encoding virulence factors exhibited this transcriptional response including T3SS locus and ACT genes [Figure 2B (cluster c); Table 5; Supplementary Table S4 (cluster c)]. As expected, the transcriptional profile of these genes indicated that they are not regulated by RisAS and phosphorylation of RisA during modulating (Bvg-) conditions and they do not require RisAS and phosphorylation of RisA for modulation or the expected transcriptional response as vags.

A total of 133 vags exhibited no significant change in gene expression in *ΔrisA*, *ΔrisAS*, and *risAD60N* compared to WT in modulating (Bvg-) conditions and lost an expected vag transcriptional response, i.e., they were not upregulated in non-modulating (Bvg+) conditions when growth in non-modulating (Bvg+) conditions was compared to modulating (Bvg-) conditions for each of these mutants [Figure 2B (cluster d); Table 5; Supplementary Table S4 (cluster d)]. Genes that exhibited this transcriptional response include *bdcB* (GGDEF domain) and 22 genes encoding predicted transcriptional regulators [Figure 2B (cluster d); Table 5; Supplementary Table S4 (cluster d)]. The transcriptional profile of these genes indicated that while they are not regulated by RisAS and phosphorylation of RisA during modulating (Bvg-) conditions, they require RisAS and phosphorylation of RisA for modulation or the expected transcriptional response as vags.

### RisS in non-modulating (Bvg+) conditions

**Figure 3:**
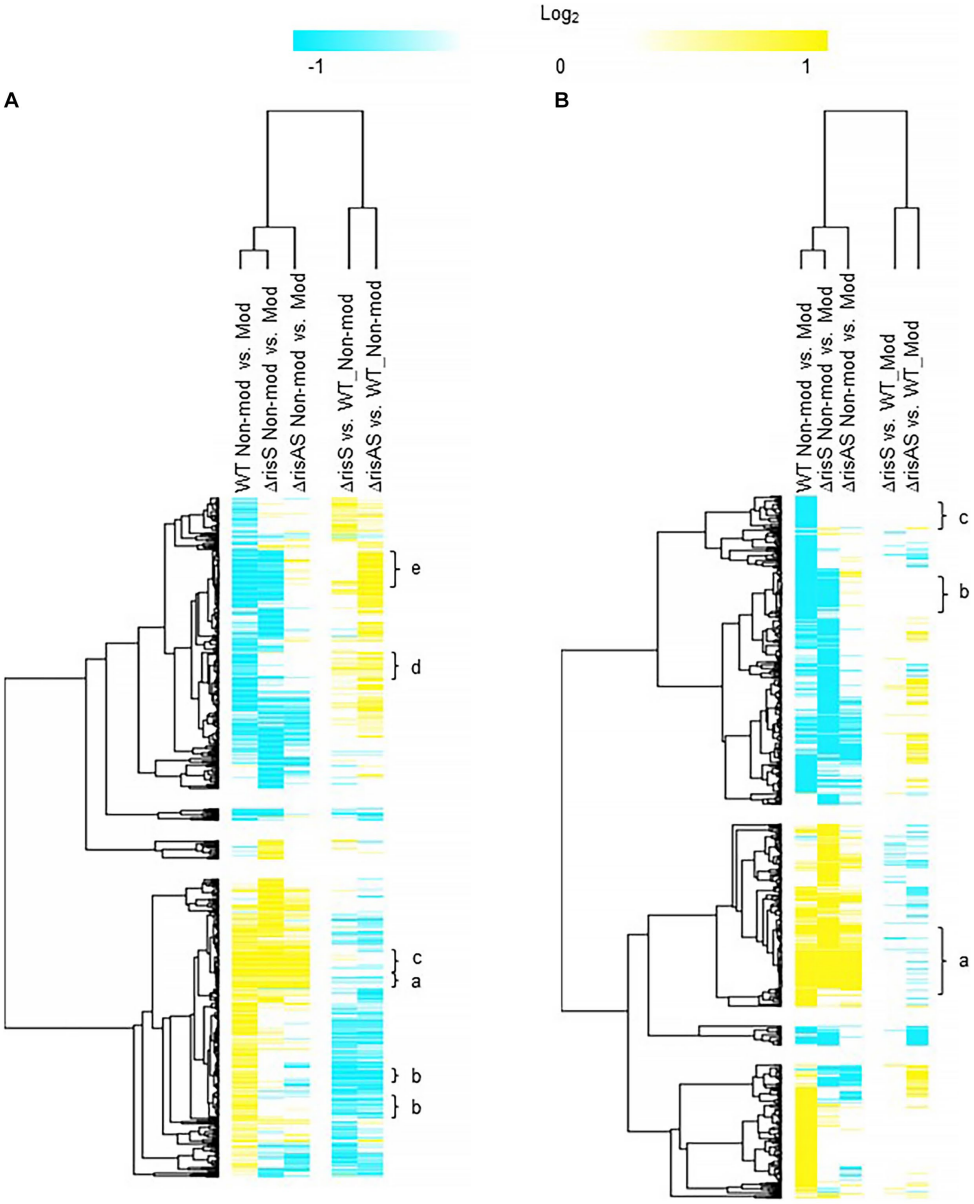
Hierarchical cluster heatmap displaying the transcriptional response of *ΔrisS* in non-modulating (Bvg+) conditions and modulating (Bvg-) conditions. **(A)** Hierarchical cluster heatmap displaying the transcriptional response of *ΔrisS* in non-modulating (Bvg+) conditions. **(B)** Hierarchical cluster heatmap displaying the transcriptional response of *ΔrisS* in modulating (Bvg-) conditions. Hierarchical clustering of the expression values was carried out using a distance method metric of 1-Pearson correlation and complete linkage. Clustering was performed on columns and rows. Dendrograms are on the left side and on top of the heat map. Isolates names and specific comparisons are provided at the top of the heat map. All expression profiles of genes are in row and are represented using the color scale at top. Yellow, indicates increased gene expression; blue, decreased gene expression; white, no significant change in gene expression. Letters represent clusters of genes with similar transcription pattern.

When *ΔrisS* and *ΔrisAS* was compared to WT in non-modulating (Bvg+) conditions, 394 genes were upregulated in *ΔrisAS* compared to WT, while only 65 genes were upregulated in both *ΔrisS* and *ΔrisAS* compared to WT in non-modulating (Bvg+) conditions (Figure 3A; Supplementary Table S5). In contrast, 390 were downregulated in *ΔrisAS* compared to WT, while 212 genes were downregulated in both *ΔrisS* and *ΔrisAS* compared to WT in non-modulating (Bvg+) conditions (Figure 3A; Supplementary Table S5). The observed differences in the number of genes differentially regulated between the comparison of each mutant to WT highlights the differences in regulation between RisA and RisS and allows for the requirement of either RisA or RisAS for repression during non-modulating (Bvg+) conditions to be determined.

Of the 212 genes downregulated in both *ΔrisS* and *ΔrisAS* compared to WT in non-modulating (Bvg+) conditions, 125 were vags (Figure 3A; Supplementary Table S5). These genes can be further divided into vags that either maintained (cluster a) or lost (cluster b) an expected vag transcriptional response, i.e., upregulation in non-modulating (Bvg+) conditions. Only five T3SS genes, *bcrH1*, *btc22*, *bsp22*, *bopN*, and CJ015_16290, were upregulated in non-modulating (Bvg+) conditions and therefore maintained an expected vag transcriptional response when growth in non-modulating (Bvg+) conditions was compared to modulating (Bvg-) conditions for each of these mutants. The transcriptional response of these genes indicated that the expression of these genes is dependent on both RisA and RisS in non-modulating (Bvg+) conditions, however, RisAS is not required for maintaining the expected transcriptional response as vags [Figure 3A (cluster a); Table 6; Supplementary Table S5 (cluster a)]. Seventy five vags, including a gene encoding a fimbrial protein and 16 genes encoding transcriptional regulators, lost a vag transcriptional response, i.e., they were not upregulated in non-modulating (Bvg+) conditions when growth in non-modulating (Bvg+) conditions was compared to modulating (Bvg-) conditions for each of these mutants [Figure 3A (cluster b); Table 6; Supplementary Table S5 (cluster b)]. The transcriptional response of these genes indicated that both RisA and RisS are required for full activation in non-modulating (Bvg+) conditions and for maintaining the expected transcriptional response as vags.

**Table 6:** *ΔrisS* in non-modulating (Bvg+) conditions.

Cluster	WT Non-Mod vs. Mod	ΔrisS Non-Mod vs. Mod	ΔrisAS Non-Mod vs. Mod	ΔrisS vs. WT Non-Mod	ΔrisAS vs. WT Non-Mod	RisA/RisS requirement for repression in Bvg + conditions	Description	Gene
a	Up	Up	Up	Down	Down		Vags dependent both RisA and RisS for full expression in non-modulating (Bvg+) conditions, but do not require RisA or RisS for maintaining an expected vag transcriptional response	*bopN* (T3SS)
b	Up	NS	NS	Down	Down		Vags dependent both RisA and RisS for full expression in non-modulating (Bvg+) conditions and for maintaining an expected vag transcriptional response	CJ015_08855 (fimbrial protein)
c	Up	Up	Up	NS	NS		Vags not regulated by RisAS during non-modulating (Bvg+) conditions and do not require RisA or RisS for maintaining an expected vag transcriptional response	FHA and PRN
d	Down	NS	NS	Up	Up	Requires both RisA and RisS	Vrgs that require both RisA and RisS for maintaining an expected vrg transcriptional response and require both RisA and RisS for repression in non-modulating (Bvg+) conditions	*tonB* (siderophore)
d	Down	NS	NS	NS	Up	Requires only RisA	Vrgs that require both RisA and RisS for maintaining an expected vrg transcriptional response and require only RisA for repression in in non-modulating (Bvg+) conditions	*livG* (amino acid transport)
e	Down	Down	NS	Up	Up	Requires both RisA and RisS	Vrgs that do not require RisA or RisS for maintaining an expected vrg transcriptional response and require both RisA and RisS for repression in in non-modulating (Bvg+) conditions	*flaA* (flagellar protein)
e	Down	Down	NS	NS	Up	Requires only RisA	Vrgs that do not require RisAS for maintaining an expected vrg transcriptional response and require only RisA for modulation or repression in non-modulating (Bvg+) conditions	*lapD* (c-di-GMP receptor)

Transcriptional response of genes within clusters identified from the comparisons used to evaluate the differential expression of the Δ*risS* mutant in non-modulating (Bvg-) conditions. Yellow, indicates genes upregulated (Up) (log2fc > 1); blue, indicates genes downregulated (Down) (log2fc < −1); white, no significant change or NS in gene expression (log2fc < 1, but > −1).

Further focusing on vags, 98 exhibited no significant change in gene expression in both *ΔrisS* and *ΔrisAS* compared to WT in non-modulating (Bvg+) conditions and were upregulated in non-modulating (Bvg+) conditions and therefore maintained an expected vag transcriptional response when growth in non-modulating (Bvg+) conditions was compared to modulating (Bvg-) conditions for each of these mutants [Figure 3A (cluster c); Table 6; Supplementary Table S5 (cluster c)]. The majority of genes encoding virulence factors exhibited this transcriptional response including PRN, FHA, and genes located within the T3SS locus [Figure 3A (cluster c); Table 6; Supplementary Table S5 (cluster c)]. As expected, the transcriptional response of these genes suggests that they are not regulated by RisAS during non-modulating (Bvg+) conditions, and they do not require RisAS for maintaining the expected transcriptional response as vags.

When evaluating expression profiles of the vrgs, 204 were upregulated in *ΔrisAS* compared to WT in non-modulating (Bvg+) conditions. These genes can be further divided into vrgs that either exhibited no significant change in gene expression or were upregulated in *ΔrisS* compared to WT in non-modulating (Bvg+) conditions, allowing for the requirement of either RisA alone or RisAS for repression during non-modulating (Bvg+) conditions to be determined. Of the 204 vrgs upregulated in *ΔrisAS* compared to WT in non-modulating (Bvg+) conditions, 162 of these genes exhibited no significant change in gene expression in *ΔrisS* compared to WT in non-modulating (Bvg+) conditions, indicating that they require RisA but not RisS for repression in non-modulating (Bvg+) conditions (Supplementary Table S5). Genes that exhibited this transcription response include *livG* and genes containing predicted domains related to cyclic di-GMP metabolism domains, *lapD* CJ015_18520 (EAL, GGDEF domain), CJ015_09185 (EAL domain), CJ015_16540 (HD-GYP domain), CJ015_16560 (PilZ, PilZN domain) (Table 6; Supplementary Table S5). In contrast, 40 vrgs were upregulated in both *ΔrisS* and *ΔrisAS* compared to WT in non-modulating (Bvg+) conditions, indicating that they require both RisA and RisS for repression in in non-modulating (Bvg+) conditions (Table 6; Supplementary Table S5). Genes that exhibited this transcription profile include *flaA*, and other chemotaxis/ motility genes (Table 6; Supplementary Table S5).

Further evaluating expression profiles of the vrgs upregulated in *ΔrisAS* compared to WT in non-modulating (Bvg+) conditions, 63 lost an expected vrg transcriptional response, i.e., they were not repressed or downregulated in non-modulating (Bvg+) conditions when growth in non-modulating (Bvg+) conditions was compared to modulating (Bvg-) conditions for each of these mutants, indicating that they require RisAS for modulation [Figure 3A (cluster d); Table 6; Supplementary Table S5 (cluster d)]. Genes that exhibited this transcription profile include *tonB* and *livG* [Figure 3A (cluster d); Table 6; Supplementary Table S5 (cluster d)]. Thirty vrgs were upregulated in *ΔrisAS* compared to WT in non-modulating (Bvg+) conditions, and were repressed or downregulated in non-modulating (Bvg+) conditions and therefore maintained an expected vrg transcriptional response when growth in non-modulating (Bvg+) conditions was compared to modulating (Bvg-) conditions for each of these mutants, indicating that they do not require RisAS for modulation [Figure 3A (cluster e); Table 6; Supplementary Table S5 (cluster e)]. Genes that exhibited this transcription profile include *lapD,* and *flaA* [Figure 3A (cluster e); Table 6; Supplementary Table S5 (cluster e)].

### RisS in modulating (Bvg-) conditions

When *ΔrisS* and *ΔrisAS* was compared to WT in modulating (Bvg-) conditions, 190 genes were upregulated in *ΔrisAS* compared to WT, while only 6 genes were upregulated in both *ΔrisS* and *ΔrisAS* compared to WT in modulating (Bvg-) conditions (Figure 3; Supplementary Table S6). In contrast, 196 were downregulated in *ΔrisAS* compared to WT, while only 14 genes were downregulated in both *ΔrisS* and *ΔrisAS* was compared to WT in modulating (Bvg-) conditions (Figure 3B; Supplementary Table S6).

Focusing on vags, 102 exhibited no significant change in both *ΔrisS* and *ΔrisAS* compared to WT in modulating (Bvg-) conditions and were upregulated in non-modulating (Bvg+) conditions and therefore maintained an expected vag transcriptional response when growth in non-modulating (Bvg+) conditions was compared to modulating (Bvg-) conditions for each of these mutants [Figure 3B (cluster a); Table 7; Supplementary Table S6 (cluster a)]. Many genes encoding virulence factors exhibited this transcriptional response include *bopN* and other T3SS genes. As expected, the transcriptional profile of these genes indicated that they are not regulated by RisAS during modulating (Bvg-) conditions, and they do not require RisAS for maintaining an expected vag transcriptional response.

**Table 7:** *ΔrisS* in modulating (Bvg-) conditions.

Cluster	WT Non-Mod vs. Mod	ΔrisS Non-Mod vs. Mod	ΔrisAS Non-Mod vs. Mod	ΔrisS vs. WT Mod	ΔrisAS vs. WT Mod	Description	Gene
**a**	Up	Up	Up	NS	NS	Vags not regulated by RisAS during modulating (Bvg-) conditions and do not require RisA or RisS for maintaining an expected vag transcriptional response	*bopN* (T3SS)
**b**	Down	Down	Down	NS	NS	Vrgs not regulated by RisAS in modulating (Bvg-) and do not require RisA or RisS for maintaining an expected vrg transcriptional response	*ureE* (urease protein)
**c**	Down	NS	NS	NS	NS	Vrgs not regulated by RisAS in modulating (Bvg-) conditions but require RisA and RisS for maintaining an expected vrg transcriptional response	*tonB* (siderophore)

Transcriptional response of genes within clusters identified from comparisons used to evaluate the differential expression of the Δ*risS* mutant in non-modulating (Bvg-) conditions. Yellow, indicates genes upregulated (Up) (log2fc > 1); blue, indicates genes downregulated (Down) (log2fc < −1); white, no significant change or NS in gene expression (log2fc < 1, but > −1).

When evaluating expression profiles of the vrgs, 305 exhibited no significant change in *ΔrisS* and *ΔrisAS* compared to WT in modulating (Bvg-) conditions (Supplementary Table S6). These genes can be further divided into vrgs that either maintained (cluster b) or lost (cluster c) an expected vrg transcriptional response, i.e., repressed or downregulated in non-modulating (Bvg+) conditions when growth in non-modulating (Bvg+) conditions was compared to modulating (Bvg-) conditions for each of these mutants. Forty two vrgs were downregulated in non-modulating (Bvg+) conditions and therefore maintained a vrg transcriptional response, indicating that these genes they are not regulated by RisAS in modulating (Bvg-) conditions and RisAS is not required for modulation [Figure 3B (cluster b); Table 7; Supplementary Table S6 (cluster b)]. Genes that exhibited this transcriptional response include *ureE* [Figure 3B (cluster b); Supplementary Table S6 (cluster b)]. In contrast, 140 vrgs lost an expected vrg transcriptional response, i.e., they were not repressed or downregulated in non-modulating (Bvg+) conditions when growth in non-modulating (Bvg+) conditions was compared to modulating (Bvg-) conditions for each of these mutants [Figure 3B (cluster c); Table 7; Supplementary Table S6 (cluster c)]. Genes that exhibited this transcription profile include *tonB* [Figure 3B (cluster c); Table 7; Supplementary Table S6 (cluster c)]. The transcriptional profile of these genes indicated that while they are not regulated by RisAS in modulating (Bvg-) conditions, they require RisA and RisS for modulation.

### BvgAS, along with BvgR, RisA, and the phosphorylation of RisA all serve a role in regulating intracellular concentrations of c-di-GMP and GMP

**Figure 4:**
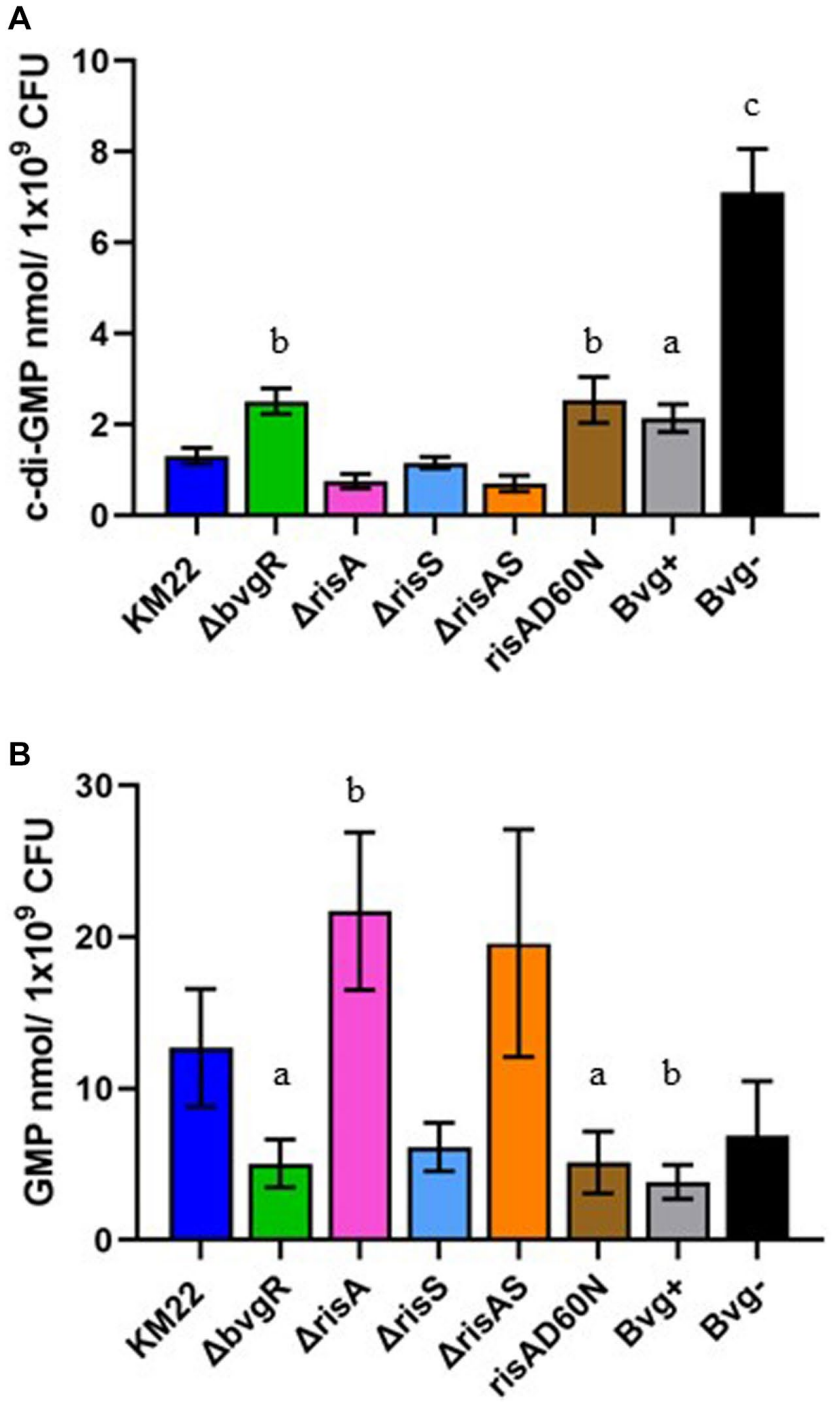
Total intracellular c-di-GMP and GMP concentrations in *B. bronchiseptica* strains. Extracted c-di-GMP **(A)** and GMP **(B)** from indicated *B. bronchiseptica* strains (x-axis) was quantified by analyzed by LC–MS/MS and normalized to CFU (y-axis). Bars represent geometric means ± SD for each *B. bronchiseptica* strain. Statistical difference between specified strain and KM22 is indicated as **(A)** *p* ≤ 0.05, **(B)** *p* ≤ 0.01, and **(C)** and *p* < 0.0001 by ordinary one-way analysis of variance (ANOVA) with a Dunnett’s multiple comparison post-test.

To evaluate the role of BvgR, RisA, phosphorylation of RisA, and RisS in regulating total intracellular concentrations of c-di-GMP and GMP, whole-cell nucleotide extractions of KM22, *ΔbvgR*, *ΔrisA*, *ΔrisAS*, *ΔrisS*, *risAD60N* mutants, along with Bvg^+^ phase-locked and Bvg¯phase-locked strains, were analyzed by LC–MS/MS for the quantification of c-di-GMP and GMP (Figure 4). Significantly increased intracellular concentrations of c-di-GMP were observed in both the Bvg + and Bvg-strains compared to KM22 (Figure 4A). Significantly increased levels of c-di-GMP were also observed in the *ΔbvgR* and *risAD60N* mutants compared to KM22 (Figure 4A). In contrast, similar levels of c-di-GMP compared to WT were observed in the *ΔrisA*, *ΔrisAS*, *ΔrisS* mutants (Figure 4A).

When the total intracellular concentrations of GMP were quantified, significantly lower levels of GMP were observed in the Bvg + strain and in the *ΔbvgR* and *risAD60N* mutants compared to KM22 (Figure 4B). Conversely, significantly increased levels of GMP were observed in the *ΔrisA* mutant (Figure 4B). These data indicate that the BvgAS system, along with BvgR, RisA, and the phosphorylation of RisA all serve a role in regulating intracellular concentrations of c-di-GMP and GMP.

### BvgR and RisA temporally regulate motility

**Figure 5:**
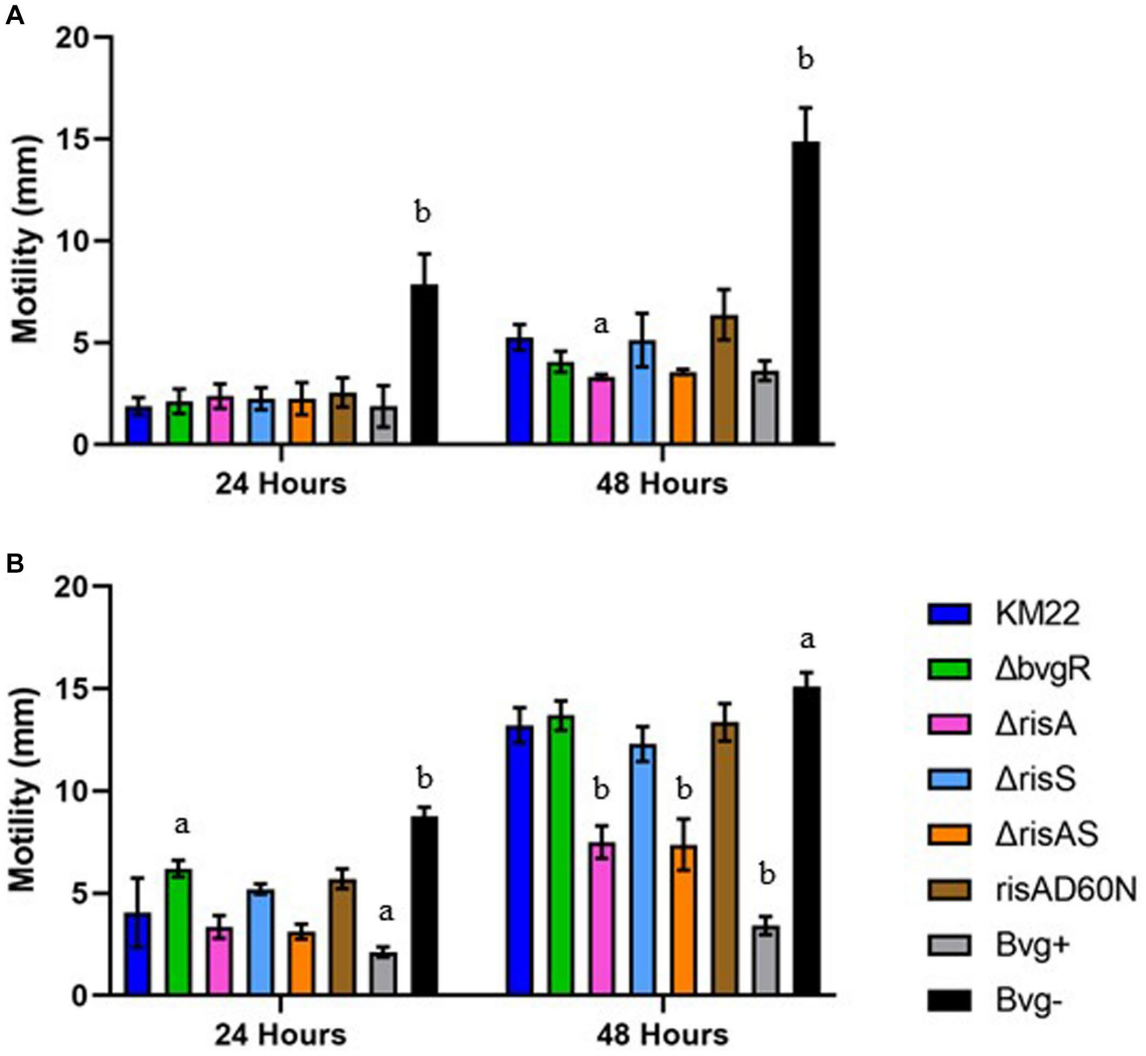
Soft-agar motility of *B. bronchiseptica* strains. Swimming motility using a soft agar (0.35%) motility assay in non-modulating (Bvg+) conditions **(A)** and in modulating (Bvg-) conditions with 50 mM MgSO_4_ **(B)**. *B. bronchiseptica* strains listed in right panel. Motile organisms display outward migration from the point of inoculation and the diameter of the migration zone (y-axis) was measured at 24 and 48 h (x-axis). Bars represent geometric means ± SD for each *B. bronchiseptica* strain. Statistical difference between specified strain and KM22 for each time point is indicated as (a) *p* ≤ 0.05 and (b) *p* ≤ 0.01 by two-way analysis of variance (ANOVA) with a Dunnett’s multiple comparisons test.

To investigate the contribution of BvgR, RisAS, and phosphorylation of RisA to motility, we compared *ΔbvgR*, *ΔrisA*, *ΔrisAS*, *ΔrisS*, *risAD60N* mutants, along with Bvg^+^ phase-locked and Bvg¯phase-locked strains, to wild-type KM22 for the ability to exhibit swimming motility during growth in soft agar plates in non-modulating (Bvg+) conditions and in soft agar plates supplemented with 50 mM MgSO_4_ to elicit modulating (Bvg-) conditions. A previously characterized Bvg^+^ phase-locked strain, which is non-motile and does not produce flagella, and a previously characterized Bvg-phase-locked strain, which is motile and produces flagella, where included as controls (Nicholson et al., 2012a). The diameter of the migration zone was measured at 24 and 48 h. As expected, the Bvg-phase-locked strain exhibited significantly increased swimming motility compared to KM22 at both 24 and 48 h during both non-modulating (Bvg+) and modulating (Bvg-) conditions (Figures 5A,B). Apart from the Bvg-phase-locked strain, all strains exhibited little to no motility at 24 h during non-modulating (Bvg+) conditions (Figure 5A). At 48 h the *ΔrisA* mutant exhibited significantly decreased motility compared to KM22 during non-modulating (Bvg+) conditions (Figure 5A). During modulating (Bvg-) conditions the *ΔbvgR* mutant exhibited significantly increased motility compared to KM22, while no significant difference in motility for the *ΔrisA*, *ΔrisAS*, *ΔrisS*, *risAD60N* mutants compared to KM22 was observed at 24 h (Figure 5B). In contrast, both the *ΔrisA* and *ΔrisAS* mutants exhibited significantly decreased motility compared to KM22, while the *ΔbvgR*, *ΔrisS*, and risAD60N mutants exhibited similar motility compared to KM22 at 48 h during modulating (Bvg-) conditions (Figure 5B). These results demonstrate that BvgR represses motility at 24 h but not 48 h, suggesting a temporal repression of motility. These results also suggest a temporal requirement for RisA for swimming motility given that no significant difference in motility was observed for the *ΔrisA* and *ΔrisAS* mutants compared to KM22 was observed at 24 h, however, both the *ΔrisA* and *ΔrisAS* mutants exhibited significantly decreased motility compared to KM22 at 48 h.

### BvgR, RisA, and RisS is required for biofilm formation, but phosphorylation of RisA is not required for biofilm formation

**Figure 6:**
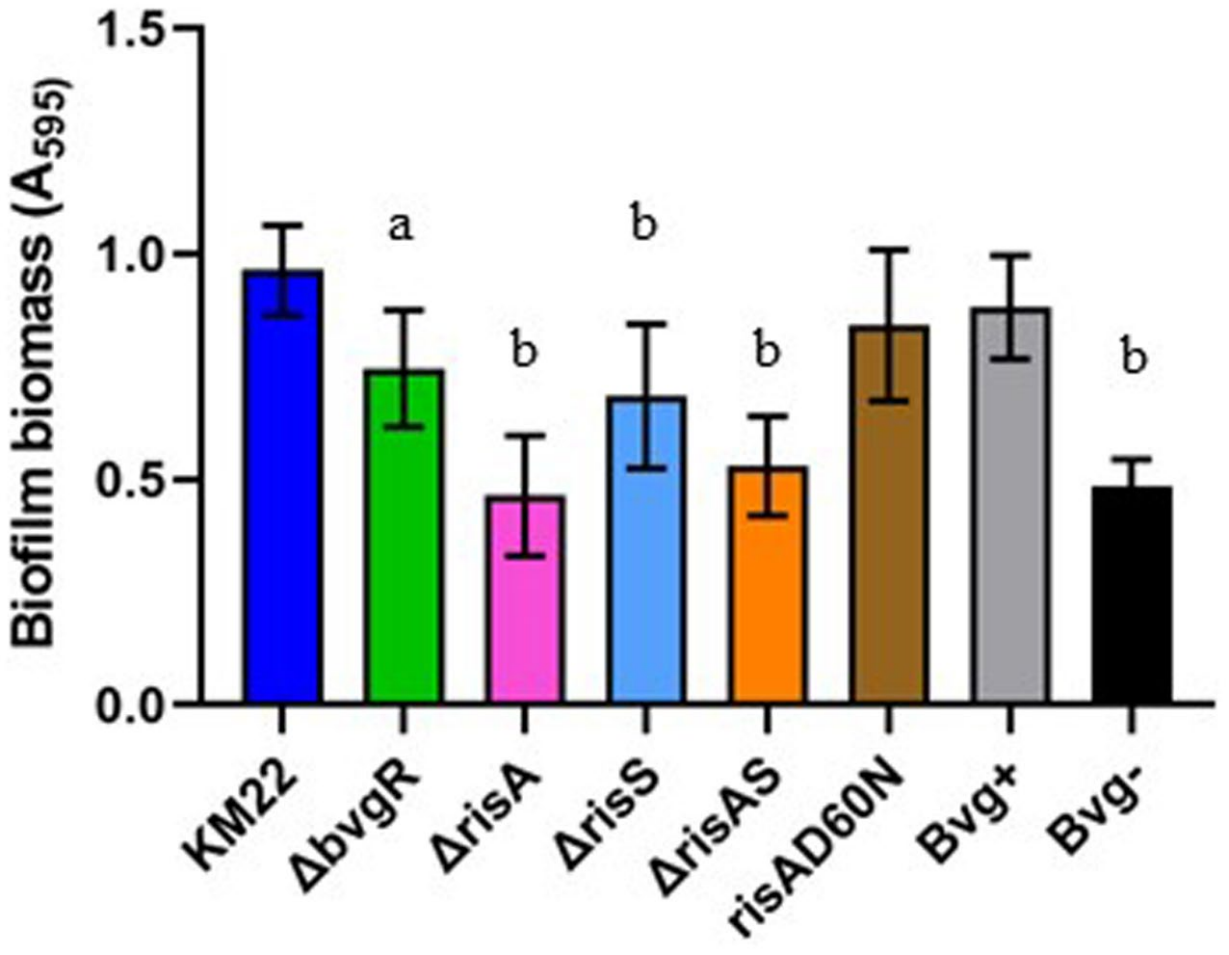
Biofilm forming capacity of *B. bronchiseptica* strains. *B. bronchiseptica* strains (x-axis) were grown statically for 24 h. Biofilm formation was quantified by standard microtiter assays and measuring the absorbance at 595 nm (y-axis). Bars represent geometric means ± SD for each *B. bronchiseptica* strain. Statistical difference between specified strain and KM22 is indicated as (a) *p* ≤ 0.05 and (b) *p* ≤ 0.0001 by one-way analysis of variance (ANOVA) with a Dunnett’s multiple comparison post-test.

To investigate the contribution of BvgR, RisA, phosphorylation of RisA, and RisS in biofilm formation, we compared *ΔbvgR*, *ΔrisA*, *ΔrisAS*, *ΔrisS*, *risAD60N* mutants, along with Bvg^+^ phase-locked and Bvg¯phase-locked strains, to KM22 for the ability to form biofilms (Figure 6). Biofilm formation was quantitated by standard microtiter crystal violet assays at 24 h and compared to KM22. Mutants *ΔbvgR*, *ΔrisA*, *ΔrisAS* mutants, along with the Bvg-control strain, all exhibited a significantly decreased ability to form biofilms compared to KM22 (Figure 6). These results suggest that BvgR and RisAS are required for biofilm formation. Additionally, these results suggest that while RisA is required for biofilm formation, phosphorylation of RisA is not required for biofilm formation.

## Discussion

In this report we assessed the global transcriptome of wildtype KM22, *ΔbvgR*, *ΔrisA*, *risAD60N*, and *ΔrisAS* under both modulating and non-modulating conditions. We identified an array of vags and vrgs that either were and were not regulated by BvgR, RisA, phosphorylation of RisA, and/or RisS. We identified vags that required BvgR, and/or RisAS and/or phosphorylation of RisA for both full activation in Bvg + conditions as well as for maintaining their expected transcriptional response as vags during modulating (Bvg-) conditions. When *ΔbvgR* was compared to KM22 in non-modulating (Bvg+) conditions, 34 vags were upregulated in KM22 and identified as requiring BvgR for full expression in non-modulating (Bvg+) conditions. Genes that exhibited this transcriptional response include genes located within the T3SS locus and known virulence factors such as PRN and ACT. The transcription response of these genes differed from most virulence factors, such as *fhaB*, *fimABCD*, *fimN*, *fim2*, and genes located within the T3SS locus that were equally expressed in both *ΔbvgR* and KM22 in non-modulating (Bvg+) conditions and identified as not regulated by BvgR. A similar difference in transcriptional response among vags was observed when *ΔrisA*, *ΔrisAS*, and *risAD60N* was compared to KM22 in non-modulating (Bvg+) conditions in which 82 vags, including genes encoding most known virulence factors, were observed as equally expressed in *ΔrisA*, *ΔrisAS*, and *risAD60N* compared to KM22. In contrast, 76 vags were downregulated in *ΔrisA*, *ΔrisAS*, and *risAD60N* when compared to KM22 in non-modulating (Bvg+) conditions indicating that RisAS and phosphorylation of RisA is required for full expression in non-modulating (Bvg+) conditions and for modulation or the maintaining an expected vag transcriptional response. Eight vags, including five T3SS genes, were downregulated in *ΔrisA*, *ΔrisAS*, and *risAD60N* compared to KM22 in non-modulating, but maintained an expected vag transcriptional response. When *ΔrisA*, *ΔrisAS*, and *risAD60N* was compared to KM22 in modulating (Bvg-) conditions, 133 vags lost the expected vag transcriptional response when growth in non-modulating (Bvg+) conditions was compared to modulating (Bvg-) conditions. This transcriptional response indicated that while they are not regulated by RisAS and phosphorylation of RisA during modulating (Bvg-) conditions, they require RisAS and phosphorylation of RisA for maintaining an expected vag transcriptional response.

Coutte et al. recently reported the regulatory pathways governed by BvgR, RisA, and phosphorylation of RisA, and RisK, the specific *B. pertussis* cognate histidine kinase of RisA, in *B. pertussis*, and additionally identified 18 vags that required RisA and phosphorylation of RisA for maintaining their expected transcriptional response as vags (Coutte et al., 2016). However, the number of vags identified as regulated by BvgR, RisAS, and phosphorylation of RisA in *B. bronchispetica* reported here far exceeds the number observed in *B. pertussis*. Differential expression of vags has been previously described, including the differential expression of genes located within the T3SS locus, and demonstrated to affect the corresponding phenotypes (Nicholson et al., 2009). A proposed biological relevance for the differential expression of vags observed here and in *B. pertussis* is that vags have been demonstrated to be expressed either early in the infectious process or late in the infectious process (Veal-Carr and Stibitz, 2005). This proposal by Coutte et al. highlights the importance of the regulation of vag expression by BvgR, RisAS, and phosphorylation of RisA in the correct time during the infectious process (Coutte et al., 2016).

Focusing on vrgs, vrgs repressed by BvgR could be divided further into vrgs that either required or did not require BvgR for modulation. Similarly, vrgs identified as regulated by RisA could be divided further into vrgs that required or did not require RisA phosphorylation, in addition to vrgs that required RisA alone or vrgs that required both RisA and RisS. For example, the capsule locus and the chemotaxis/ motility locus are two vrg gene loci that exhibited differential regulation by RisA. In the absence of *risA*, the capsule locus was not expressed in modulating (Bvg-) conditions, while the chemotaxis/ motility locus was expressed in modulating (Bvg-) conditions. Additionally, the capsule locus required RisA for expression in modulating (Bvg-) conditions, with some genes within the locus also requiring the phosphorylated form of RisA, while the chemotaxis/ motility locus required both RisA and RisS for expression in modulating (Bvg-) conditions. These results contrast with the Coutte et al., that reported for *B. pertussis* the expression for almost all vrgs required both RisA and RisA phosphorylation in Bvg-conditions (Coutte et al., 2016). These divergent transcriptional responses add to the other previously key differences between *B. bronchispetica* and *B. pertussis* despite the close relativeness and evolutionary link.

When intracellular concentrations of c-di-GMP were quantitated, significantly increased intracellular concentrations of c-di-GMP were detected in both the ΔbvgR and risAD60N mutants compared to KM22. Given that BvgR contains a conserved EAL sequence found in diguanylate phosphodiesterases that are involved in the degradation of c-di-GMP, increased intracellular concentrations of c-di-GMP demonstrates that BvgR is serves a role in c-di-GMP degradation. The data additionally suggest that phosphorylated RisA serves a role in c-di-GMP degradation. Correspondingly, both lower levels of GMP were observed for both the *ΔbvgR* and *risAD60N* mutants compared to KM22. Significantly increased levels of GMP were detected in *ΔrisA* and *ΔrisAS* compared to KM22, suggesting unphosphorylated RisA alone serves a role in the accumulation of intracellular GMP.

In general, genes located within the chemotaxis/ motility locus exhibited the largest differential gene expression observed from all the mutants. When we assessed motility, temporal differences were observed. Specifically, during modulating (Bvg-) conditions BvgR repressed motility at 24 h but not 48 h, while RisA alone and not the phosphorylated form of RisA, was required for motility at 48 h but not 24 h. The observed temporal regulation highlights the strict regulation of flagella production. Stringent control over flagella production is required for *B. bronchispetica* biofilm formation (Nicholson et al., 2012b). While modulating (Bvg-) conditions and flagella production is favored during initial stages of biofilm formation, flagella repression is absolutely required for the development of mature and structured biofilms (Nicholson et al., 2012b). We found BvgR and RisAS to be required for biofilm formation. Additionally, we found that while RisA is required for biofilm formation, phosphorylation of RisA is not required for biofilm formation. Overexpression of a *Pseudomonas aeruginosa* protein with DGC activity in *B. bronchispetica* has been shown to increase intracellular levels of c-di-GMP, increase static biofilm formation, and reduce motility (Sisti et al., 2013). While both BvgR and RisA were found to impact biofilm and motility phenotypes, the impact did not lead to an expected increase in biofilm formation or a decrease in motility at both time points. Both phenotypes are regulated by complex temporal pathways, which likely account for the different phenotypic outcomes.

Both BvgR and phosphorylated RisA were found to serve a role in c-di-GMP degradation as well as global gene regulation for *B. bronchiseptica.* While the functional contribution of vrgs to the infectious process of *B. bronchiseptica* remains unknown, a prominent hypothesis is that vrgs promote survival in aerosols generated by coughing and therefore increase transmission success. Collectively, the data reported here help to provide specific gene targets to aid in investigating the role of c-di-GMP intracellular levels on transmission success.

## Data availability statement

The datasets presented in this study can be found in online repositories. The names of the repository/repositories and accession number(s) can be found in the article/Supplementary material.

## Author contributions

TN: Conceptualization, Data curation, Formal analysis, Funding acquisition, Investigation, Methodology, Project administration, Resources, Supervision, Writing – original draft, Writing – review & editing. UW: Data curation, Investigation, Methodology, Resources, Writing – review & editing. DF: Data curation, Formal analysis, Investigation, Methodology, Resources, Software, Visualization, Writing – original draft, Writing – review & editing. QC: Investigation, Methodology, Resources, Writing – review & editing. LM: Formal analysis, Investigation, Methodology, Resources, Software, Writing – original draft, Writing – review & editing. TM: Conceptualization, Writing – review & editing. SS: Conceptualization, Investigation, Methodology, Resources, Writing – review & editing.

## Footnotes

^1^https://doi.org/10.48550/arXiv.1303.3997

^2^https://clue.io/morpheus

^3^https://usegalaxy.eu
