# Supplementary figures and images for: The contribution of BvgR, RisA, and RisS to global gene regulation, intracellular cyclic-di-GMP levels, motility, and biofilm formation in Bordetella bronchiseptica
Source: Front Microbiol. 2024 Mar 7;15:1305097. doi: 10.3389/fmicb.2024.1305097 (PMC10955343; doi:10.3389/fmicb.2024.1305097)

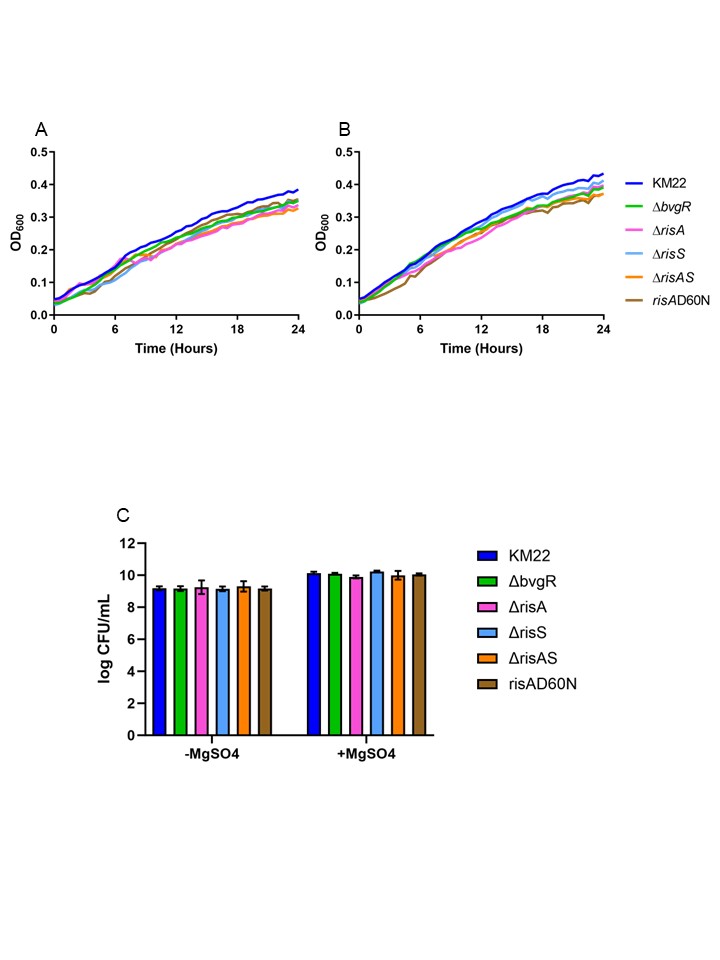

Supplement: Supplementary file 7 [file Image_1.JPEG]

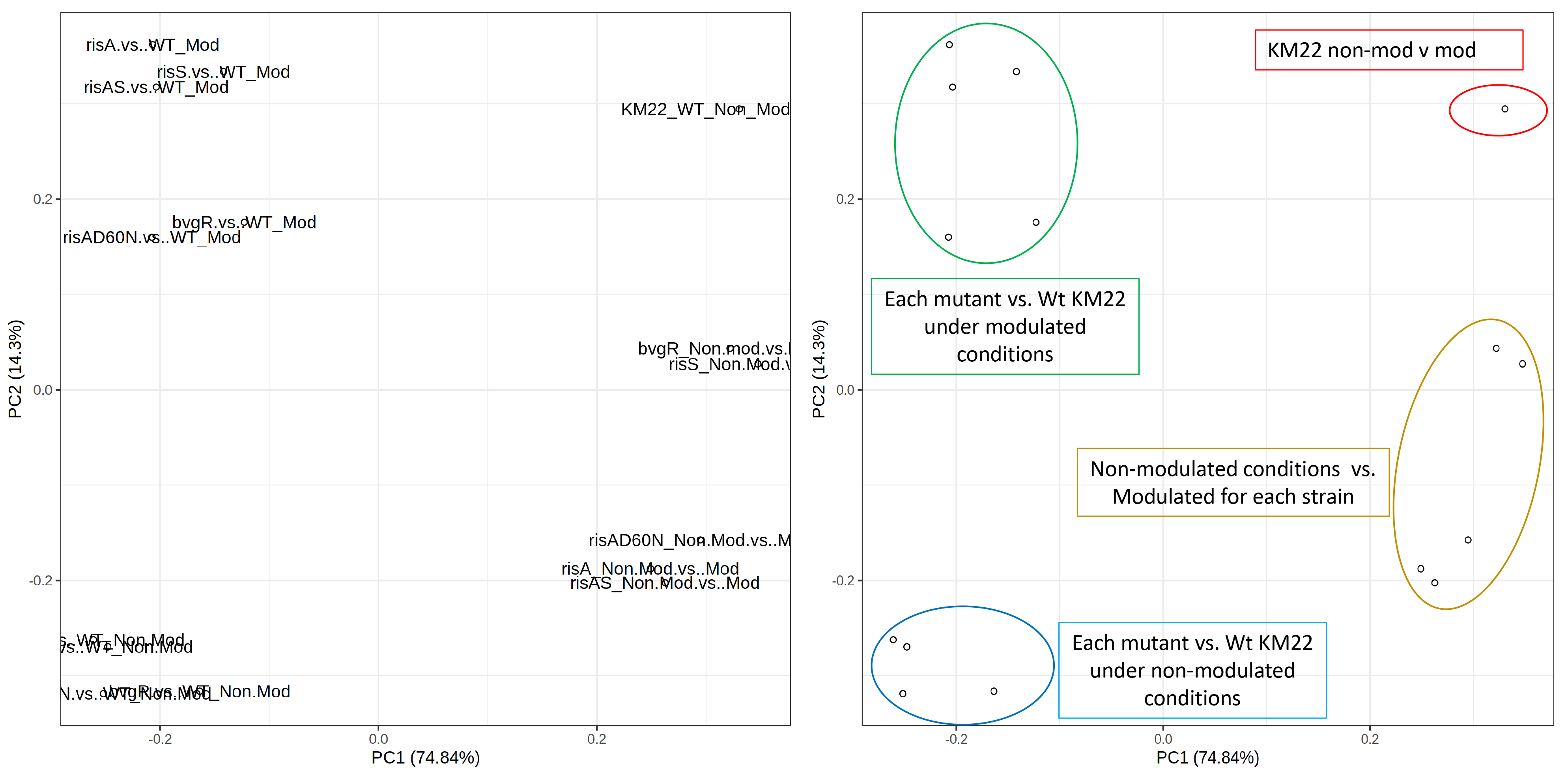

Supplement: Supplementary file 8 [file Image_2.JPEG]
