# Supplementary material for: The contribution of BvgR, RisA, and RisS to global gene regulation, intracellular cyclic-di-GMP levels, motility, and biofilm formation in Bordetella bronchiseptica
Source: Front Microbiol. 2024 Mar 7;15:1305097. doi: 10.3389/fmicb.2024.1305097 (PMC10955343; doi:10.3389/fmicb.2024.1305097)
Supplement: Supplementary file 9 [file Data_Sheet_1.pdf]

Supplemental Figure Legends

Figure S1. Growth kinetics of *B. bronchiseptica* strains. Growth curves of *B. bronchiseptica* strains listed in right panel grown in either (A) SS broth supplemented with 40µg/mL streptomycin or (B) SS broth supplemented with 40µg/mL streptomycin and 50 mM $MgSO_4$ Optical densities (OD_{600}) are plotted along the right y axis; and time, given in hours, is plotted along the x axis. All data points represent averages obtained from eight biological replicates. CFU levels for *B. bronchiseptica* strains listed in right panel after 11 hours of growth in either SS broth supplemented with 40µg/mL streptomycin (-$MgSO_4$) or SS broth supplemented with 40µg/mL streptomycin and 50 mM $MgSO_4$ (+$MgSO_4$). Y-axis indicates the mean CFU expressed as the $\log_{10}$ mean +/- the standard error (error bars).

Figure S2. PCA plot based on correlation matrix of expression values for all *Bordetella* genes in the experiment. The plot shows that the samples were positively correlated within the conditions they were treated. The treatment conditions explained over 74%. Separated sample groups based on treatments (green, yellow, and blue circles and boxes) and control samples (red circle and box). Values for correlation matrices and PCA plots performed using the R packages ggplot2 and ggfortify.

Supplemental Table 1. BvgR in non-modulating (Bvg+) conditions.

RNA-Seq data (Sheet 1); qPCR Data (sheet 2); all primers used for qPCR (Sheet3)

Supplemental Table 2. BvgR in modulating (Bvg-) conditions.

RNA-Seq data (Sheet 1)

Supplemental Table 3. RisA and phosphorylation of RisA in non-modulating (Bvg+) conditions.

RNA-Seq data (Sheet 1); qPCR Data (sheet 2)

Supplemental Table 4. RisA and phosphorylation of RisA in modulating (Bvg-) conditions.

RNA-Seq data (Sheet 1); qPCR Data (sheet 2)

Supplemental Table 5. RisS in non-modulating (Bvg+) conditions.

RNA-Seq data (Sheet 1); qPCR Data (sheet 2)

Supplemental Table 6. RisS in modulating (Bvg-) conditions

RNA-Seq data (Sheet 1); qPCR Data (sheet 2)
